# Supplementary figures and images for: Eosinophils of patients with localized and diffuse cutaneous leishmaniasis: Differential response to Leishmania mexicana, with insights into mechanisms of damage inflicted upon the parasites by eosinophils
Source: PLoS One. 2024 Feb 15;19(2):e0296887. doi: 10.1371/journal.pone.0296887 (PMC10868813; doi:10.1371/journal.pone.0296887)

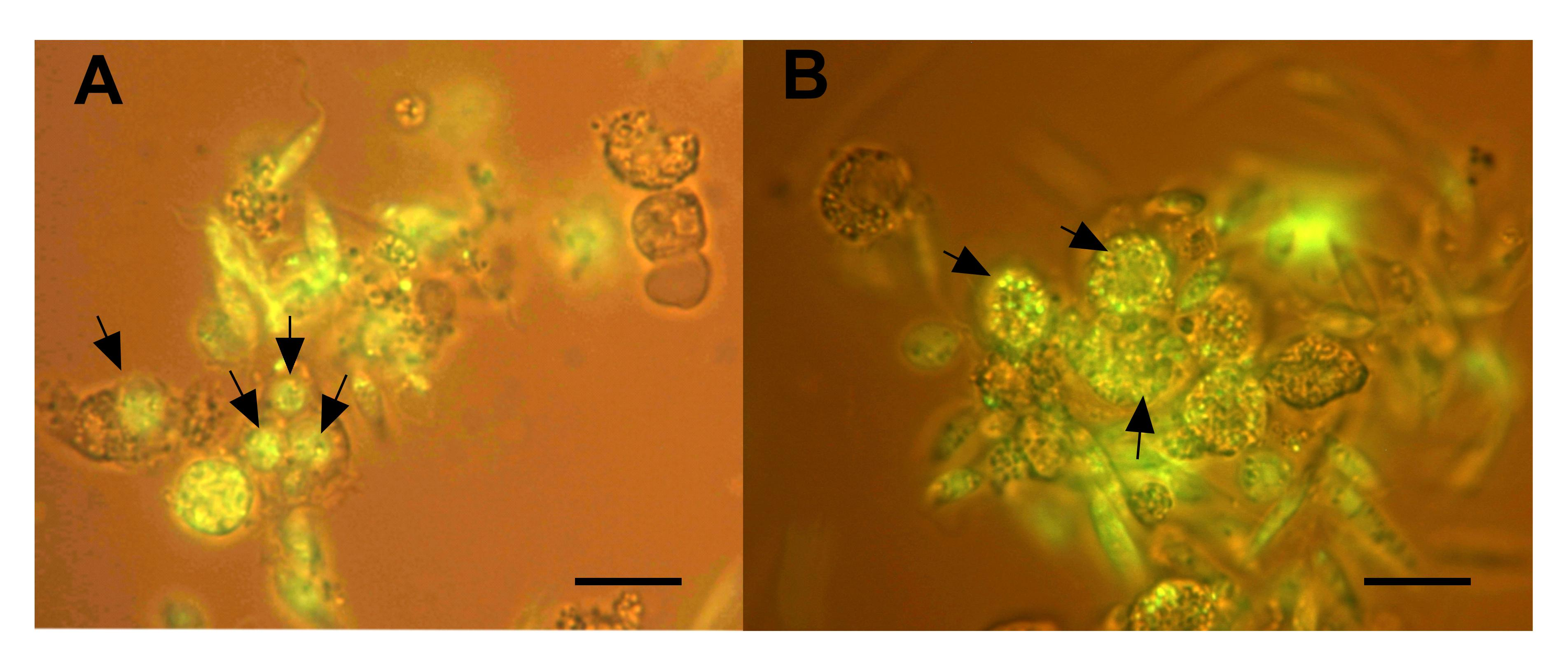

Supplement: S1 Fig — A) Parasites stained with CFDA (green color) phagocytosed by eosinophils are observed inside these cells. Black arrows show phagocytized parasites. B) Remains of degraded parasites (black arrows) are observed already inside the eosinophils (green color granules). The co-incubation ratio was 1:10 for 2 hours. Scale bar = 20 μm. (TIF) [file pone.0296887.s001.tif]

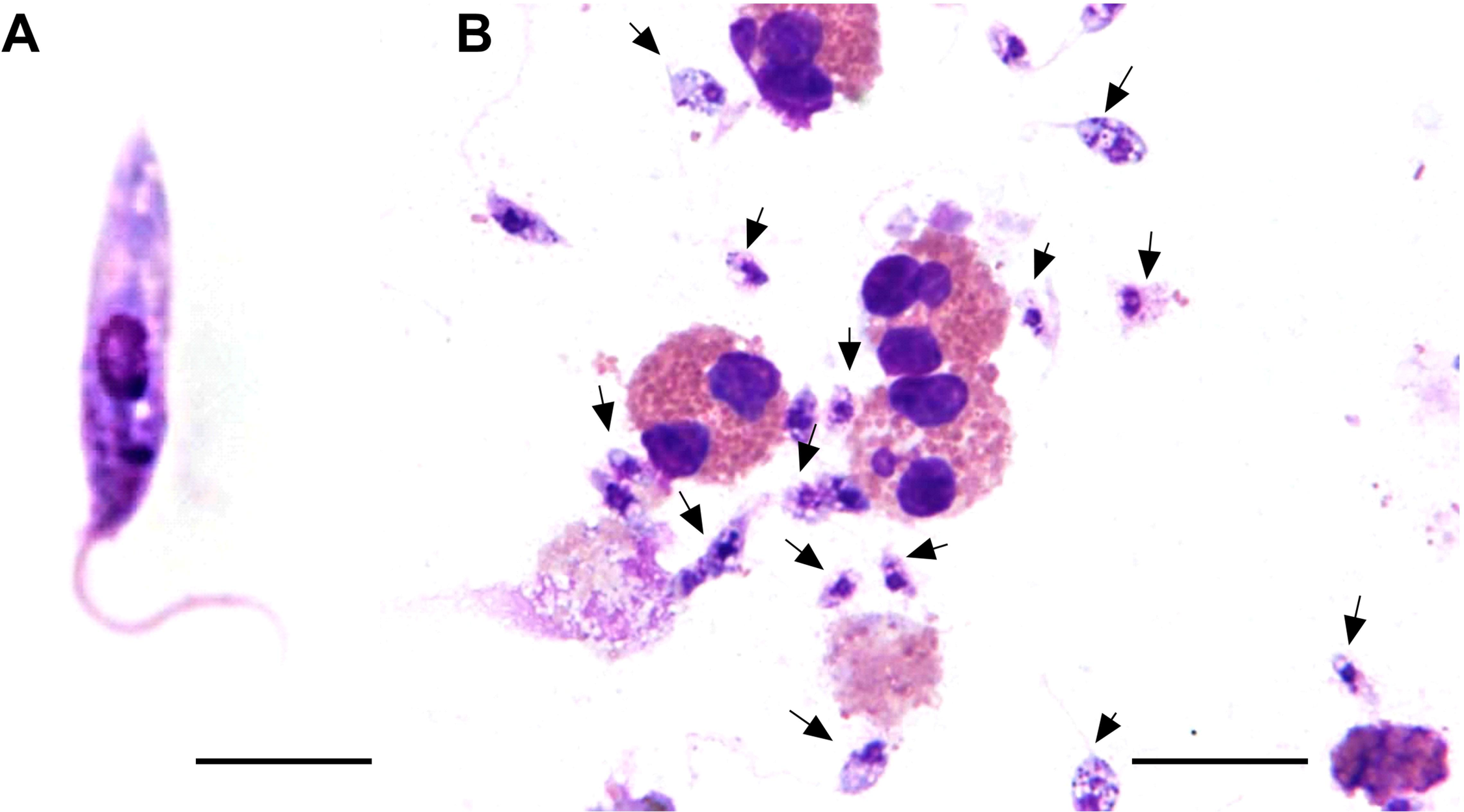

Supplement: S2 Fig — A) Normal shape and size of a viable Leishmania promastigote. B) Morphological changes in parasite size, damage to membranes, formation of small vacuoles within the parasite, and loss of flagellum were observed in parasites co-incubated with eosinophils in a 1:10 ratio for 1 hour. Black arrows show damaged parasites. Scale bar = 20 μm. (TIF) [file pone.0296887.s002.tif]
