# Supplementary material for: Eosinophils of patients with localized and diffuse cutaneous leishmaniasis: Differential response to Leishmania mexicana, with insights into mechanisms of damage inflicted upon the parasites by eosinophils
Source: PLoS One. 2024 Feb 15;19(2):e0296887. doi: 10.1371/journal.pone.0296887 (PMC10868813; doi:10.1371/journal.pone.0296887)
Supplement: Attachment — Submitted filename: PONE-D-23-05814_R1.pdf [file pone.0296887.s004.pdf]

PLOS ONE

Eosinophils of patients with localized and diffuse cutaneous leishmaniasis: differential response to Leishmania mexicana, with insights into mechanisms of damage inflicted upon the parasites by eosinophils

--Manuscript Draft--

Manuscript Number:	PONE-D-23-05814R1
Article Type:	Research Article
Full Title:	Eosinophils of patients with localized and diffuse cutaneous leishmaniasis: differential response to Leishmania mexicana, with insights into mechanisms of damage inflicted upon the parasites by eosinophils
Short Title:	Eosinophils response to Leishmania mexicana with insights into mechanisms of damage inflicted upon parasites
Corresponding Author:	Ingeborg Becker, M.D. Ph.D.<br>Universidad Nacional Autónoma de México Facultad de Medicina: Universidad Nacional Autonoma de Mexico Facultad de Medicina<br>MEXICO
Keywords:	Eosinophils, Leishmania mexicana, damage, patients, localized and diffuse leishmaniasis
Abstract:	Eosinophils are mainly associated with parasitic infections or allergic manifestations. They produce many biologically active substances, which contribute to the death of pathogens through microbicidal components degranulation and inflamatory tissue effects. In leishmaniasis, eosinophils have been found inside inflammatory infiltrated and are related to protective immunity againts the parasite. We analyzed the responses of eosinophils from patients with localized (LCL) and diffuse (DCL) cutaneous leishmaniasis, as well as of healthy subjects, to Leishmania mexicana. All patients with DCL had blood eosinophilia, as well as elevated numbers of eosinophils in non-ulcerated nodules. In contrast, only LCL patients with prolonged evolution time showed eosinophils in blood and in cutaneous ulcers. Eosinophils from DCL patients secreted significantly more IL-6, IL-8, and IL-13, compared to eosinophils from LCL patients. DCL patients also showed higher serum levels of anti-Leishmania IgG antibodies. We also showed that eosinophils from LCL and DCL patients respond to L. mexicana promastigotes with an intense oxidative burst, which was equally intense in both groups of patients, and significantly higher than in healthy subjects.<br>The coincubation of eosinophils (from donors with eosinophilia) with L. mexicana promastigotes in vitro showed various mechanisms of parasite damage related to diverse patterns of granule exocytosis: 1) localized degranulation on the parasite surface, 2) the release of cytoplasmic membrane bound “degranulation sacs” containing granules, 3) release of eosinophil extracellular traps containing DNA and granules with major basic protein.<br>In conclusion, eosinophils damage L. mexicana parasites through the release of granules by diverse mechanisms. Yet, even though DCL patients harbor abundant eosinophils in blood and tissues, their apparent inability to convey protection is possibly linked to the release of cytokines and chemokines that favor a Th2 immune response and disease progression in these patients.
Order of Authors:	Norma Salaiza-Suazo, PhD
	Roxana Porcel-Aranibar
	Isabel Cristina Cañeda-Guzmán
	Adriana Ruiz-Remigio
	Jaime Zamora-Chimal, PhD
	José Delgado-Domínguez, PhD
	Rocely Cervantes-Sarabia
	Georgina Carrada-Figueroa, PhD

Powered by Editorial Manager® and ProduXion Manager® from Aries Systems Corporation

	Baldomero Sánchez-Barragán
	Victor Javier Leal-Ascencio
	Armando Pérez-Torres, MD, PhD
	Héctor A. Rodríguez-Martínez
	Ingeborg Becker, M.D. Ph.D.

Response to Reviewers:

Reviewer #1: The article “Eosinophils of patients with localized and diffuse cutaneous leishmaniasis: differential response to Leishmania mexicana, with insights into mechanisms of damage inflicted upon the parasites by eosinophils “ by Norma Salaiza-Suazo et al., describe in several experiments the presence and response of eosinophils in LCL and DCL. Eosinophils have been neglected in the leishmaniasis contexts, probably because their significance is overshadowed by the importance of macrophages and T cells. This study address a relevant gap in the knowledge. However results here shown are rather descriptive than conclusive.

Major comments:

1) Methods section is in many points poorly described:
a) Need more specification on the eosinophil isolation: How long was centrifuged? How was done the RBC lysis? How many washes/time/g-force?

R= New information was added on lines 168-190

b) -Leishmania was cultured in the absence of antibiotics?
R=The information was added on lines 196-197.

c) -Also, in-house method for IL-8 measurement lacks important information: which capture/detection antibody was used? Please describe the protocol.

R=The information was added on lines 207-226.

d) -How was the cut-off in ELISA calculated?

R= The cut-off point information was added on lines 223-224.

e) - How was the cytometry done? What was the antibodies used and gating strategy applied?

R= Eosinophils in peripheral blood were determined by FCS and SSC by flow cytometry, this information was added on lines 133-134. The figure below represents the gating strategy.

f) -Why is the luminescence in oxidative bursts measured as Mmvolt?

R= The Luminoskan's light detector has a photomultiplier tube (photomultiplier tube), a signal acquisition and processor system, which are in a closed compartment, where all the chemiluminescence is captured by the photomultiplier tube which sends the light signal and according to the configuration of our device (Luminoskan) the maximum light signal generated in the chemiluminescence reaction is reported in mVolts.

Line 309 – 310 “These findings demonstrate that eosinophils from patients with DCL produce significantly more IL-6, IL-8, and IL-13 when exposed to L. mexicana promastigotes, compared to those of LCL patients.” Cytokine measurement should have been done with unstimulated eosinophils. This is important to be sure that the cytokine production is not due to a pre-activated status. How could the authors be sure the cytokines produced were induced by parasites rather than being already activated?
R= Unstimulated eosinophils were included in all cytokine production determinations.

Powered by Editorial Manager® and ProduXion Manager® from Aries Systems Corporation

However, they were not included in the plot because they were below the cut-off point. The information was added on lines 352-354.

Line 318-330 – To assume that eosinophils are being harmful against leishmania, the authors should show quantitatively increased death of promastigotes while in contact with Leishmania. They could use cellular death markers (Propidium iodide for example) to quantify the number of dead promastigotes in contact with eosinophil.
R= No staining with supravital dyes was performed, however, to demonstrate cell damage we are now including a new supplementary figure 2 showing Giemsa-stained viable intact parasites compared to damaged parasites incubated with eosinophils, where morphological changes and cell damage of the parasites are visible. This observation was also evidenced in the electron microscopy photographs of Figure 6, showing damage of the external membrane of the parasite.

Lines 407-409 – It is not correct to associate IL-6 with TH2 responses. IL-6 is mainly produced by myeloid cells (mostly macrophages) and this cytokine is increased during VL together with a TH2 response, independently. IL-6 is required to a TH17 response and whether their production by eosinophils is important/significant during cutaneous leishmaniasis is yet to be shown.

R= We agree and have removed the statement regarding the association of IL-6 with a Th2 response. Instead, we added the relevance of IL-6 in the Th17 response in cutaneous leishmaniasis on lines 459-464.

Line 435: The authors says that “it is noteworthy that 70% LCL and 90% DCL were positive for IgE”, although figure 3 clearly shows (as well as is described in the text) that all patients were IgG and IgE positive. Please revise this excerpt and figure.

R= We thank the reviewer and made the correction on line 488.

Lines 442 – 444 – authors must be careful to assume “an enhanced production of cytokines” in DCL patients, since they only measured eosinophil derived cytokines and this does not mean that these cytokines are in fact increased in this patients or even related to susceptibility (for example, IFN-g is associated with resistance and it is also increased during VL). The production of these cytokines by eosinophils compared to other cells (macrophages and monocytes) might be insignificant in natural conditions.

R= We thank the reviewer and have changed the text on lines 496-501.

Figure 5 - Authors claim to demonstrate phagocitozed leishmania by eosinophil but the image has bad quality. Do authors have clear evidence of phagocytosis by eosinophils (stained slides would be enough).

R= Figure 5 was modified with a better photograph in panel 5D that shows parasites phagocyted by eosinophils. Additionally, we added a new supplementary figure 1 with more photographs showing the phagocytosis and possible intracellular degradation of the parasite.

Authors should discuss why an innate immune cell would respond differently in DCL and LCL. Which possible mechanisms would allow eos from DCL to be more prone to respond to promastigotes than LCL?

R= We now added a possible explanation for the differential response between LCL and DCL patients on lines 452-457, including a new reference (30) where differences in genetic background between both patients have been reported.

Line 449: “their release of the cytokines IL-6, IL-8 and IL-13 promotes disease severity”: the data here shown do not support this statement.

R= We agree with the reviewer and changed the statement on lines 503-505.

Powered by Editorial Manager® and ProduXion Manager® from Aries Systems Corporation

	Reviewer #2: The authors report on an important and up-to-now largely neglected aspect of a cellular repsonse to Leishmania infection. This study certainly will be of great interest.<br><br>Major concerns:<br><br>- The statistical method employed should be detailled in the respective Fig. legends. The authors might want to use multivariate testing.<br><br>R= The objective of the work was to compare the response between eosinophils of LCL and DCL patients and for this approach we decided use to the Mann–Whitney U-test.<br>New information was added in Figure legends 1, 2 and 5, detailing the statistical significance of the figures.<br><br><br>- The Fig. 5 - 7 should be analyzed using a readout that allows statistical analysis. Otherwise these findings seem very anectodal.<br><br>R= We agree and modified Figure 5, adding the panel 5E, that quantitatively describes the % of degranulation induced by parasite incubation. This result was also included in line 375-376.<br><br><br>- The authors should demonstrate a by FACS that their MACS-based enrichment of eosinophils worked.<br><br>R= We don´t have a FACS analysis of the enrichment of eosinophils, but include for the reviewer the figure below that shows a Giemsa stain of peripheral blood cells from patients before (A, B) and after the MACS-based enrichment (C, D). The purity of eosinophils was >99%. The information was described on lines 190-191. This purity was analyzed by a microscopical analysis of the characteristic acidophilic granules and the polymorphic nuclei as markers of eosinophils.<br><br><br>Figure 1. Giemsa stain of eosinophil MACS-based enrichment. A and B show peripherical blood cells from eosinophilic patients. C and D show eosinophil enrichment. Scale bar = 20 μm.<br><br>I am sorry, but I could include the image to this text to show the enriched eosinophils.
**Additional Information:**	
**Question**	**Response**
**Financial Disclosure**<br><br>Enter a financial disclosure statement that describes the sources of funding for the work included in this submission. Review the submission guidelines for detailed requirements. View published research articles from *PLOS ONE* for specific examples.<br><br>This statement is required for submission and **will appear in the published article** if the submission is accepted. Please make sure it is accurate.	This work was supported by UNAM-PAPIIT IG201221 and CONACyT-Fronteras 6682. The funders had no role in study design, data collection and analysis, decision to publish or preparation of the manuscript.

**Unfunded studies**<br>Enter: *The author(s) received no specific funding for this work.*<br><br>**Funded studies**<br>Enter a statement with the following details:<br>• Initials of the authors who received each award<br>• Grant numbers awarded to each author<br>• The full name of each funder<br>• URL of each funder website<br>• Did the sponsors or funders play any role in the study design, data collection and analysis, decision to publish, or preparation of the manuscript?<br>• **NO** - Include this sentence at the end of your statement: *The funders had no role in study design, data collection and analysis, decision to publish, or preparation of the manuscript.*<br>• **YES** - Specify the role(s) played.<br><br>* typeset	
**Competing Interests**<br><br>Use the instructions below to enter a competing interest statement for this submission. On behalf of all authors, disclose any competing interests that could be perceived to bias this work—acknowledging all financial support and any other relevant financial or non-financial competing interests.<br><br>This statement is required for submission and **will appear in the published article** if the submission is accepted. Please make sure it is accurate and that any funding sources listed in your Funding Information later in the submission form are also declared in your Financial Disclosure statement.<br><br>View published research articles from *PLOS ONE* for specific examples.	The authors have declared that no competing interests exist.

Powered by Editorial Manager® and ProduXion Manager® from Aries Systems Corporation

<table>
<tr>
<td>

NO authors have competing interests

Enter: *The authors have declared that no competing interests exist.*

Authors with competing interests

Enter competing interest details beginning with this statement:

I have read the journal's policy and the authors of this manuscript have the following competing interests: [insert competing interests here]

* typeset

</td>
<td></td>
</tr>
<tr>
<td>

Ethics Statement

Enter an ethics statement for this submission. This statement is required if the study involved:

- Human participants
- Human specimens or tissue
- Vertebrate animals or cephalopods
- Vertebrate embryos or tissues
- Field research

Write "N/A" if the submission does not require an ethics statement.

General guidance is provided below. Consult the submission guidelines for detailed instructions. **Make sure that all information entered here is included in the Methods section of the manuscript.**

</td>
<td>

The study was reviewed and approved by the Ethics and Research Committees of the Facultad de Medicina, UNAM (Universidad Nacional Autónoma de México) FM/DI/088/2017 and guidelines established by the Ministry of Health in Mexico were strictly followed. All patients and healthy donors (controls) were informed and signed a written consent to participate in the study.

</td>
</tr>
</table>

Powered by Editorial Manager® and ProduXion Manager® from Aries Systems Corporation

**Format for specific study types**<br><br>**Human Subject Research (involving human participants and/or tissue)**<br>• Give the name of the institutional review board or ethics committee that approved the study<br>• Include the approval number and/or a statement indicating approval of this research<br>• Indicate the form of consent obtained (written/oral) or the reason that consent was not obtained (e.g. the data were analyzed anonymously)<br><br>**Animal Research (involving vertebrate animals, embryos or tissues)**<br>• Provide the name of the Institutional Animal Care and Use Committee (IACUC) or other relevant ethics board that reviewed the study protocol, and indicate whether they approved this research or granted a formal waiver of ethical approval<br>• Include an approval number if one was obtained<br>• If the study involved *non-human primates*, add *additional details* about animal welfare and steps taken to ameliorate suffering<br>• If anesthesia, euthanasia, or any kind of animal sacrifice is part of the study, include briefly which substances and/or methods were applied<br><br>**Field Research**<br><br>Include the following details if this study involves the collection of plant, animal, or other materials from a natural setting:<br>• Field permit number<br>• Name of the institution or relevant body that granted permission	
**Data Availability**<br><br>Authors are required to make all data underlying the findings described fully available, without restriction, and from the time of publication. PLOS allows rare exceptions to address legal and ethical concerns. See the PLOS Data Policy and FAQ for detailed information.	Yes - all data are fully available without restriction

A Data Availability Statement describing where the data can be found is required at submission. Your answers to this question constitute the Data Availability Statement and **will be published in the article**, if accepted.<br><br>**Important:** Stating 'data available on request from the author' is not sufficient. If your data are only available upon request, select 'No' for the first question and explain your exceptional situation in the text box.<br><br>Do the authors confirm that all data underlying the findings described in their manuscript are fully available without restriction?	
**Describe where the data may be found in full sentences. If you are copying our sample text, replace any instances of XXX with the appropriate details.**<br><br>• If the data are **held or will be held in a public repository**, include URLs, accession numbers or DOIs. If this information will only be available after acceptance, indicate this by ticking the box below. For example: *All XXX files are available from the XXX database (accession number(s) XXX, XXX.*).<br>• If the data are all contained **within the manuscript and/or Supporting Information files**, enter the following: *All relevant data are within the manuscript and its Supporting Information files.*<br>• If neither of these applies but you are able to provide **details of access elsewhere**, with or without limitations, please do so. For example:<br><br>*Data cannot be shared publicly because of [XXX]. Data are available from the XXX Institutional Data Access / Ethics Committee (contact via XXX) for researchers who meet the criteria for access to confidential data.*<br><br>*The data underlying the results presented in the study are available from (include the name of the third party*	All relevant data are within the manuscript and its Supporting Information files.

and contact information or URL).<br>• This text is appropriate if the data are owned by a third party and authors do not have permission to share the data.<br><br>* typeset	
Additional data availability information:	

Manuscript Click here to access/download;Manuscript;Manuscript.docx

Eosinophils of patients with localized and diffuse cutaneous leishmaniasis: differential response to *Leishmania mexicana*, with insights into mechanisms of damage inflicted upon the parasites by eosinophils

Norma Salaiza-Suazo[1], Roxana Porcel-Aranibar[1], Isabel Cristina Cañeda-Guzmán[1], Adriana Ruiz-Remigio[1], Jaime Zamora-Chimal[1], José Delgado-Domínguez[1], Rocely Cervantes-Sarabia[1], Georgina Carrada-Figueroa[2], Baldomero Sánchez-Barragán[3], Victor Javier Leal-Ascencio[4], Armando Pérez-Torres[5], Héctor A. Rodríguez-Martínez[1], Ingeborg Becker[1*]

[1]Unidad de Investigación en Medicina Experimental, Facultad de Medicina, Universidad Nacional Autónoma de México, Ciudad de México 04510, México.

[2]División Académica de Ciencias de la Salud, Universidad Juárez Autónoma de Tabasco (UJAT), Tabasco, México

[3]Escuela de Medicina, Universidad Juárez Autónoma de Tabasco (UJAT), Tabasco, México

[4] Hospital Regional de Alta Especialidad Dr. Juan Graham, Secretaría de Salud del Estado de Tabasco, Villahermosa, Tabasco, México

[5] Departamento de Biología Celular y Tisular, Facultad de Medicina, Universidad Nacional Autónoma de México, Ciudad de México, México

*Corresponding author

Ingeborg Becker

Tel. -52-55-56232674

e-mail: becker@unam.mx

Abstract

Eosinophils are mainly associated with parasitic infections and allergic manifestations. They produce many biologically active substances, that contribute to the destruction of pathogens through the degranulation of microbicidal components and inflammatory tissue effects. In leishmaniasis, eosinophils have been found within inflammatory infiltrate with protective immunity against the parasite. We analyzed the responses of eosinophils from patients with localized (LCL) and diffuse (DCL) cutaneous leishmaniasis, as well as from healthy subjects, when exposed to *Leishmania mexicana.*

All DCL patients exhibited blood eosinophilia, along with elevated eosinophil counts in non-ulcerated nodules. In contrast, only LCL patients with prolonged disease progression showed eosinophils in their blood and cutaneous ulcers. Eosinophils from DCL patients secreted significantly higher levels of IL-6, IL-8, and IL-13, compared to eosinophils from LCL patients. Additionally, DCL patients displayed higher serum levels of anti-*Leishmania* IgG antibodies. We also demonstrated that eosinophils from both LCL and DCL patients responded to *L. mexicana* promastigotes with a robust oxidative burst, which was equally intense in both patient groups and significantly higher than in healthy subjects.

Coincubation of eosinophils (from donors with eosinophilia) with *L. mexicana* promastigotes *in vitro* revealed various mechanisms of parasite damage associated with different patterns of granule exocytosis: 1) localized degranulation on the parasite surface, 2) the release of cytoplasmic membrane-bound “degranulation sacs” containing granules, 3) release of eosinophil extracellular traps containing DNA and granules with major basic protein.

In conclusion, eosinophils damage *L. mexicana* parasites through the release of granules via diverse mechanisms. However, despite DCL patients having abundant eosinophils in their blood and tissues, their apparent inability to provide protection may linked to the release of cytokines and chemokines that promote a Th2 immune response and disease progression in these patients.

Introduction

In humans, *Leishmania mexicana* can cause localized cutaneous leishmaniasis (LCL) and diffuse cutaneous leishmaniasis (DCL). The most common clinical form of the disease occurring in over 90% of the cases is LCL. It is characterized by ulcers at the sites of parasite inoculation, accompanied by a robust cellular immune response and a scarce number of parasites within the lesions. Patients with LCL generally experience a relatively benign clinical course [1]. Less than 1% progress to DCL, which is characterized by multiple non-ulcerated nodules containing heavily parasitized macrophages that cover large areas of the body. These patients exhibit an ineffective cell-mediated immune response against *Leishmania* parasites, leading to uncontrolled and potentially fatal progression [2]. In Mexico, 500-1000 new cases of cutaneous leishmaniasis are diagnosed every year [3].

In the early stages of infection, the inflammatory infiltrate includes neutrophils, macrophages, and eosinophils. Under normal conditions, eosinophils constitute only 1–5% of circulating leukocytes, although they can also be found in tissues as resident cells [4].

Eosinophils are well equipped to combat pathogens, such as helminths, and their numbers increase during infection [5]. Eosinophils play key roles in immune

modulation, tissue remodeling, and repair, primarily through the secretion and degranulation of inflammatory mediators, including cytokines and chemokines. However, their effector mechanisms can also inadvertently damage bystander cells and tissues [5, 6].

During the acute phase of leishmaniasis, eosinophils can be present in skin lesions, often alongside mast cells. Furthermore, a close association between eosinophils and parasitized macrophages has been reported in chronic lesions, suggesting that eosinophils may contribute to parasite destruction through cooperation with macrophages [7]. Tissue eosinophilia has been observed in DCL patients, with evidence of parasitized and lysed eosinophils and dispersion of their granules near parasitized macrophages [8]. Additionally, both in the mouse model of leishmaniasis and in humans with chronic phases of the disease, eosinophils are frequently found in granulomas [9, 10, 11]. One of the mechanisms through which eosinophils assist in controlling the parasite is the production of hydrogen peroxide, which has a detrimental effect on *Leishmania amazonensis* [12]. However, the precise physiological role of eosinophils in leishmaniasis remains incompletely understood.

In this study, we conducted a comparative analysis of the *in vitro* response of eosinophils isolated from LCL and DCL patients, following their co-incubation with *L. mexicana.* Our analysis included the measurement of IL-6, IL8 and IL-13 production, as well as assessment of their oxidative burst.

Additionally, we co-incubated eosinophils from non-*Leishmania* eosinophilic subjects with *L. mexicana* promastigotes and analyzed their defense mechanisms against the parasites. Our findings reveal that eosinophils employ various defense

mechanisms when confronted with *L. Mexicana.* These mechanisms include phagocytosis, contact degranulation, the release of granules directly onto the parasite, expulsion of “degranulation sacs” containing granules surrounded by a membrane, which are released into the immediate vicinity of the parasites, and the release of eosinophil extracellular traps (EETs) containing DNA and granules rich in major basic protein (MBP). All of these mechanisms result in irreversible damage to the parasite. Our data provide new insights into the innate defense mechanisms exerted by eosinophils against *L mexicana*, as well as differences in the eosinophil response between LCL and DCL patients.

Materials and methods

Ethics Statement

The study received approval from the Ethics and Research Committees of the Facultad de Medicina, UNAM (Universidad Nacional Autónoma de México) with reference FM/DI/088/2017. We adhered strictly to the guidelines established by the Ministry of Health in Mexico. All participants and healthy donors (controls), were informed and provided written consent to participate in the study.

Patients

The number of participants varied across different study sections and these numbers are specified in each respective section. Patients diagnosed with LCL and DCL were residents of the State of Tabasco, an endemic area for *L. mexicana* in southeastern Mexico. All patients received anti-*Leishmania* treatment with pentavalent antimonials, as mandated by National Health Authorities. LCL patients

presented active lesions with relatively few parasites. In contrast, DCL patients exhibited multiple nodular lesions characterized by heavily parasitized macrophages. The severity of DCL cases ranged from patients with nodules confined to the upper body to those with nodules affecting the entire body, limbs and oral and nasal mucosae. *Leishmania* parasites were detected in smears created from biopsy punches taken from the lesions, stained using Giemsa and subjected to histopathological analysis with hematoxylin and eosin (H&E) staining. For *in vitro* experiments, eosinophils from individuals with eosinophilia (n=8) of unknown origin who sought care at the allergy ward in the Hospital General de Mexico, were utilized. Only individuals with eosinophilia exceeding 6% were included in this study.

Peripheral blood eosinophils were quantified via flow cytometry, using FCS and SSC parameters, in patients with LCL (n=30) and DCL (n=4). Punch skin biopsies (Stiefel Laboratories, Inc., Coral Gables, FL, USA) were obtained from the lesions of LCL (n= 35) and DCL patients (n= 5), using 2% xylocaine as a local anesthesia. The biopsy specimens were fixed in 10% formalin and embedded in paraffin (Sigma-Aldrich, St. Louis, Mo, USA), cut into 4 µm thick sections, and stained with H&E, to evaluate eosinophil infiltration. Eosinophils were counted in eight images of each tissue, with a final area corresponding to 1 mm^2, using an Axio imager M1 microscope equipped with an MRc5 digital camera and Axiovision 4.8 software (Carl Zeiss, Industrielle Messtechnik GmbH, Oberkochen Germany).

Detection of IgE and IgG in Human Serum Against Leishmania mexicana Antigens

We determined IgE and IgG levels against *L. mexicana* antigens through ELISA in LCL (n=8) and DCL patients (n=8), as well as in healthy controls (n=8). Each sample was analyzed in triplicate. Briefly, 96-well EIA/RIA plates (Corning, NY, USA) were coated with 0.6 µg/100 µl of lysed promastigotes in each well and incubated for 1 hour. The solution was removed and the plates were blocked with 200 µl of 0.5% casein for 1 hour. After blocking, 100 µl of diluted (1:5) sera from leishmaniasis patients or healthy donors were added and incubated overnight at 4ºC. The plates were washed with PBS-Tween. For IgE detection, 100 µl of rabbit anti-human IgE (Dako, Agilent Technologies, Sta Clara, Ca, USA) (1:500) was added and incubated for 1 hour, followed by washing and the addition of 100 µl of biotin goat anti-rabbit IgG (1:8000) (Thermo Fisher Scientific, Waltham, Ma, USA) for 30 minutes. After washing, 100 µl of peroxidase-conjugated streptavidin (Zymed) (1:2500) was added and incubated for 30 min. For IgG detection, 100 µl of peroxidase-conjugated goat anti-human IgG (Zymed) antibody (1:4000) was added to the plates and incubated for 1 hour. Following washing,100 µl of TMB (Tetramethylbenzidine) peroxidase substrate (Becton Dickinson, NJ, USA) was added for 20 minutes. The reaction was halted with 100 µl of 1 M phosphoric acid. Absorbance was measured at 450 nm using an ELISA microplate reader (BIO-TEK).

Eosinophil Isolation

Human eosinophils were obtained from peripheral blood samples of patients with LCL (n=30) and DCL (n=4). Cells were isolated through gradient centrifugation using Ficoll Hypaque (Sigma-Aldrich, St. Louis, Mo, USA). The blood was diluted

1:2 with sterile PBS pH7.4 and layered over 20 mL Ficoll-hypaque (density ρ = 1.077 g/mL) in a 50mL conical tube. Centrifugation was performed at 600×g for 30 minutes at 20 °C using a swinging bucket rotor. The subsequent steps involved removing and discarding the plasma. Mononuclear cells were located at the plasma:Ficoll interface, and granulocytes and erythrocytes were in the pellet. The pellet was resuspended in 1× Red Blood Cell Lysis Solution, and the complete 50 mL conical tube was filled with lysis solution. Incubation occurred for 10 minutes on ice, followed by centrifugation at 300×g for 10 minutes at 20 °C. The supernatant was carefully removed entirely. Cells were washed cells by adding 50 mL of buffer, and the cell number was determined. The cell suspension was centrifuged at 300×g for 10 minutes. The supernatant was aspirated completely, and the cells were resuspended in 50 µl of buffer per $5x10^7$ cells. To this suspension, 50 µl of anti-CD16 conjugated microbeads (Miltenyi Biotec, Bergisch-Gladbach, Germany) were added per $5x10^7$ cells, and incubation occurred for 30 minutes at 4-8 °C. Cells were washed by adding 1-2 mL of buffer per 10^7 cells, followed by centrifugation at 300×g for 10 minutes. The supernatant was discarded, and the cells were resuspended to a final volume of up to 10^8 cells in 500 µL of buffer. The suspension was then placed in a purification column, which had been previously washed with buffer and positioned in the magnetic field of a MACS separator. Unlabeled cells, including eosinophils, were obtained by depletion of magnetically labeled cells. The total effluent containing the unlabeled cell fraction harbored the enriched eosinophil fraction. The purity and viability of eosinophils were >99%, as determined by Giemsa staining and by Trypan blue exclusion, respectively.

Leishmania mexicana Promastigotes Culture

L. mexicana promastigotes were cultured in blood agar (NNN medium) overlaid with Schneider's *Drosophila* medium (Life Technologies), supplemented with 10% heat inactivated FBS (Biowest, Riverside, MO, USA) and 1% antibiotics (penicillin/streptomycin (Gibco, Life Technologies) at 28°C. Parasites were subcultured every 3-4 days and grown to a density of 1 x 10^7/ml.

Cytokine Measurement

The impact of *L. mexicana* on the production of IL-6, IL-8, and IL-13 by eosinophils from LCL (n=7) and DCL (n=7) patients, as well as healthy controls (n=7) was analyzed as follows: 1 x 10^6 cells were incubated for 18 hours at 37 °C and 5% CO_2 with 10x10^6 promastigotes in 1 ml of RPMI-1640 medium, supplemented with 10% heat-inactivated FBS. The cell-free supernatants from the cultures were collected and the concentrations of the cytokines were determined using human IL-6 and IL-13 Quantikine ELISA Kits (R&D Systems Minneapolis, MN, USA). IL-8 was analyzed by a standard sandwich ELISA. Briefly, 96-well flat-bottom microtiter plates (Costar, Corning, NY, USA) were coated with 1 µg/ml unconjugated anti-cytokine capture antibodies (Purified mouse anti-human IL-8 monoclonal antibody, Clone G265-5, PharMingen) diluted in 0.1 M Na2HPO4 (pH 9) and incubated overnight at 4°C. The plates were blocked with PBS (pH 7.4) supplemented with 0.5% casein dissolved in 0.1 N NaOH. Cell-free supernatants and recombinant hIL-8 standard (PharMingen) were incubated in RPMI-1640 medium supplemented with 10% FBS overnight at 4 °C. Bound human IL-8 was detected using 0.5 µg/ml

biotin-labeled detection antibodies (Biotinylated mouse anti-human IL-8 monoclonal antibody, Clone G265-8, PharMingen) diluted in 1% BSA with 0.05% Tween 20 and incubated for 1 hour at room temperature. The plate was developed using AP-Streptavidin Conjugate (Life TechnologiesTM) and phosphatase substrate (0.005 mg/mL, Sigma-Aldrich). Absorbance was read at 405 nm and the IL-8 concentration of each sample was calculated by regression analysis based on a standard curve. The detection limit of this assay ranged from 15.6 to 1000 pg/ml. The cut-off point is determined from the lower detection limit, values below this limit considered negative. Unstimulated eosinophils were included in all cytokine production determinations; however, they were not included in the plot as they were below the cut-off point.

Oxidative Burst

The oxidative burst was measured by chemiluminescence immunoassay with eosinophils obtained from the same group of patients and controls as in the cytokine analysis. Briefly, $1x10^6$ eosinophils were incubated with $10x10^6$ promastigotes in 80 µl of RPMI 1640 medium, 210 µl of luminol (Sigma-Aldrich, St. Louis, Mo, USA) at a concentration of 1 mg/ml, and 60 µl AB+ serum-opsonized zymosan (12.5 mg/ml). Chemiluminescence was analyzed for 30 minutes (Luminoskan Labsystem, Finland) at 37 °C with a 550 nm filter [13, 14]. The values obtained were expressed in mVolt.

Interaction of Leishmania Promastigotes and Blood Eosinophils from Healthy Controls

The interaction between *L. mexicana* and eosinophils was analyzed using 5-carboxyfluorescein diacetate (CFDA) staining. *Leishmania* promastigotes ($10x10^6$) were washed twice with cold RPMI and stained with 5 μM CFDA (Sigma-Aldrich, St. Louis, Mo, USA) for 10 min in RPMI at 37 ºC. The parasites were washed three times with PBS at 26 °C and incubated with $1x10^6$ eosinophils for 2 hours at 37 ºC, protected from light. They were then centrifuged and resuspended in 10-20 μl of PBS and analyzed with an epifluorescent microscope [15]. Some of the smears were stained with Giemsa.

Transmission Electron Microscopy

Following careful washing with PBS, co-cultures of eosinophils ($1x10^6$) and promastigotes ($10x10^6$) were fixed in 2.5% glutaraldehyde diluted in 0.2 M cacodylate buffer, pH 7.4, for 2 hours, at 4 °C. After three washes of 10 minutes each in 0.15 M cacodylate buffer, pH 7.4 at 4 °C, the pellets of the cocultures were postfixed with 1% osmium tetroxide in 0.2 M cacodylate buffer, pH 7.4, for 30 minutes at 4 °C. Dehydration was carried out in gradually increasing concentrations of ethanol, followed by transfer to absolute toluene, using two changes of 10 minutes each. Infiltration was done with araldite 6005-toluene solution (1:1) for 24 hours at room temperature and then in pure araldite 6005 (two changes) at 60 °C for 2 hours. Specimens were embedded in pure araldite 6005 at 60 °C, for 36 h. Ultrathin sections were obtained with a diamond knife, contrasted with uranyl acetate and lead citrate, and examined using an Electron Microscope

(EM) 109 (Carl Zeiss, Germany). All reagents used were from Electron Microscopy Science, Hatfield, PA, USA.

Immunocytochemistry for Detection of Eosinophil Major Basic Protein (MBP)

For the immunocytochemical analysis, 1x 10^6 purified eosinophils were incubated in a chamber slide (Nunc Lab-Tek, Sigma-Aldrich, St. Louis, Mo, USA) for 2 hours at 37 ºC and 5% CO_2 with $10x10^6$ *L. mexicana* promastigotes in 1 ml of RPMI-1640 medium supplemented with 10% heat-inactivated FBS. After incubation, they were washed with PBS and fixed with 2% paraformaldehyde (Sigma-Aldrich, St. Louis, Mo, USA). Nonspecific antigen sites were blocked with 2% bovine serum albumin, dissolved in Tris-HCl, pH 7.6. Thereafter, they were stained with mouse anti-human MBP at a dilution of 1:100 (Santa Cruz Biotechnology, Ca, USA) for 30 minutes. After washing, eosinophils were incubated with the secondary antibody, biotin goat anti-mouse, at a dilution of 1:100 (Invitrogen, Waltham, Ma, USA) for 30 minutes, washed and incubated with streptavidin AP (Alkaline Phosphatase) at a dilution of 1:100 (Thermo Fisher Scientific, Waltham, Ma, USA) for 30 minutes. After washing, they were counterstained with hematoxylin and mounted with resin. Additionally, the incubation of eosinophils with promastigotes was also stained with DAPI in separate samples (Sigma-Aldrich, St. Louis, Mo, USA) to verify the formation of DNA nets.

Statistical Analysis

Comparisons between experimental groups and controls were performed using Mann–Whitney U-test. A value of $p < 0.05$ was considered statistically significant, using Prism 8 for Windows.

Results

Eosinophils in Peripheral Blood and Lesions of LCL and DCL Patients

Blood samples were collected from 30 LCL patients (19 males and 11 females), with a mean age of 28 years and disease durations ranging from 1.5 to 18 months (16 ± 3.98 months). Four DCL patients (three males and one female) with a mean age of 37 years (range: 20 - 45 years) and disease durations ranging from 40 - 120 months were also included. Eosinophils were quantified in peripheral blood using flow cytometry. The mean percentage of eosinophils in LCL patients was 11.1 ± 1.51 (Fig. 1A), with 60% of LCL patients exhibiting eosinophilia ranging from 5% to 32% (Fig. 1B). There were no significant correlations between the number of eosinophils in peripheral blood and disease duration, age, or gender (data not shown). Notably, all female patients had a shorter disease duration compared to male patients (Fig. 1B), with females showing an average disease evolution of 6.5 ± 1.45 months, while males had an average of 21.4 ± 5.95 months.

All DCL patients exhibited blood eosinophilia, with a mean percentage of eosinophils in the blood of 16 ± 5.8%, ranging from 7% to 34% (Fig. 1A). It is noteworthy that all LCL and DCL patients with chronic infections lasting over two years had blood eosinophilia (Fig. 1B).

Eosinophils in Lesions of LCL and DCL Patients

Skin biopsies were obtained from 35 LCL patients and 5 DCL patients. When analyzing skin lesions, significant differences in eosinophil numbers were observed between both clinical forms. Eosinophils were observed in skin lesions of all 5 DCL patients, with a mean of 14 ± 1.15 eosinophils/mm^2 (ranging from 11 to18). In contrast, only 22% of LCL patients had eosinophils in their tissues, with a mean value of 3 ± 1 eosinophils/mm^2 (ranging between 0 and 18). When comparing eosinophils in tissues between all LCL and DCL patients, a significant difference was found between the groups, with significantly higher numbers in DCL patients ($p < 0.05$) (Fig. 2). However, when specifically comparing LCL patients with tissue eosinophils to all DCL patients, no significant differences were found between the two groups of patients. LCL patients with tissue eosinophils showed an average of 13 ± 1.6 eosinophils/mm^2 (ranging between 5 and 18), while DCL patients showed an average of 14 ± 1.1 eosinophils/mm^2 (ranging between 11 and 17). Histopathological analysis of infected tissues revealed that DCL patients had loss of tissue integrity and a higher parasite load, compared to those of LCL patients (Fig. 2).

Anti-Leishmania IgE and IgG in Sera from LCL and DCL Patients

Serum levels of IgE and IgG were measured in LCL (n=8) and DCL (n=8) patients to determine the serological response to *L. mexicana*. Both antibodies were identified in all patients, with no significant differences in IgE levels between DCL (1.43 ± 0.18) and LCL patients (0.95 ± 0.19). However, both patient groups produced significantly more IgE than controls (0.167 ± 0.068) (Fig. 3A). When

comparing IgG production, DCL patients (1.73 ± 0.05) produced significantly more than LCL patients (1.31 ± 0.11), and both patient groups produced more IgG than controls (0.293 ± 0.074) (Fig. 3B).

Cytokine (IL-6, IL-13, and IL-8) Production and Oxidative Burst by Eosinophils of LCL and DCL Patients Incubated with L. mexicana Promastigotes

Our data indicate that IL-6 and IL-13 production by eosinophils from patients with DCL (n=7) was significantly higher, compared to those from LCL patients (n=7) or healthy control subjects (n=7), when incubated with *L. mexicana* promastigotes. IL-6 production by DCL eosinophils (45 ± 13.2 pg/mL) was significantly higher compared to LCL patients (7.4 ± 1 pg/mL). Control subjects did not exhibit IL-6 production (Fig. 4A).

IL-8 production by eosinophils of DCL patients (45 ± 2.6 pg/mL) was also significantly higher compared to LCL patients (29 ± 2 pg/mL) and control subjects (40 ± 1.7 pg/mL). Interestingly, eosinophils of control subjects produced nearly the same amount of IL-8 as those of DCL patients) (Fig. 4B).

In the case of IL-13 production, eosinophils of DCL patients showed a significantly higher production (1000 ± 11.5 pg/mL) compared to LCL patients and controls (50 ± 2.8 pg/mL) (Fig. 4C). These findings demonstrate that eosinophils from DCL patients produce significantly more IL-6, IL-8, and IL-13 when exposed to *L. mexicana* promastigotes compared to those of LCL patients. Unstimulated

eosinophils did not exhibit IL-6, IL-8, and I-13 cytokine production and were below the ELISA cut-off point.

The analysis of the oxidative burst in eosinophils of LCL (n=7), DCL (n=7), and healthy subjects (n=7) co-incubated with *L. mexicana* promastigotes showed that the parasite enhances the magnitude of the oxidative burst in eosinophils of both groups of patients to an equal extent, which was significantly higher compared to eosinophils form healthy controls (Fig. 4D). However, no significant differences between both clinical forms.

Phagocytosis and Lysis of Leishmania mexicana Promastigotes by Eosinophils

Microscopic analysis revealed that purified eosinophils do not release granules in the absence of parasites (Fig. 5A). When co-incubated with *L. mexicana,* eosinophils exhibited the ability to damage extracellular promastigotes by releasing cytoplasmic granules. This damaged resulted in the alteration of parasite morphology and the acquisition of a deformed phenotype (Fig. 5B). Giemsa staining further demonstrated that damage occurred when *Leishmania* was in close contact with the eosinophils, as well as when parasites were surrounded by clouds of massively released granules (Fig. 5B). Staining with CFDA revealed that *L. mexicana* promastigotes began to form clusters in the culture medium (Fig. 5C). After incubation with eosinophils, the promastigotes are phagocytosed and transformed into intracellular amastigotes, ultimately leading to their degradation (Fig. 5D). The percentage of eosinophil degranulation was significantly higher in eosinophils co-incubated with *Leishmania* (Fig.5e).

Eosinophils Release Cytoplasmic "Degranulation Sacs" with Granules in Response to Leishmania

Co-incubation of eosinophils with *L. mexicana* revealed that, in addition to diffusely releasing cytoplasmic granules near parasites, eosinophils also release cytoplasmic "degranulation sacs" containing granules with electron-dense characteristics, surrounded by a membrane (Fig. 6). Subsequently, as the cell membrane was lost, individual granules came into close contact with the parasites, leading to damage (Figs. 6A and 6B). Electron microscopy analysis further illustrated that eosinophils could shed substantial portions of their cytoplasm, including granules, in the form of "degranulation sacs" onto the parasite. This phenomenon was evidenced by the presence of submembrane microtubules and caused significant damage to the parasites. Signs of parasite damage included diffuse osmophilic areas, indicating damage to intracellular membranes (Figs. 6C and 6D).

Extracellular Traps are Induced by L. mexicana

Another leishmanicidal mechanism observed *in vitro* was the release of extracellular traps (EETs) by eosinophils, which entrapped the parasites and brought them into contact with released granules such as MPB (7A). Under resting conditions, only eosinophil nuclear DNA was stained blue (7B). However, after coincubation with *Leishmania,* eosinophils released nets containing DNA, as indicated by blue DAPI staining, which binds DNA (Fig. 7C). These DNA nets effectively trapped the parasites (Fig. 7D).

Discussion

Eosinophils, constituting 2-5% of human blood leukocytes, play a multifunctional role in defending against various infections, including parasites, viruses, bacteria, and fungi [5, 6, 16]. However, ~~Yet,~~ the understanding of eosinophils' role in patients infected with *L. ~~M~~exicana,* presenting with varying disease severity, such as localized (LCL) or diffuse (DCL) cutaneous leishmaniasis, remains limited. In our study, we observed that all DCL patients, and a subset of LCL patients with prolonged disease evolution exhibited both blood and tissue eosinophilia. Blood eosinophilia has previously been reported in *L. mexicana*-infected DCL patients and recently cured LCL patients [17], as well as in patients infected with high numbers of *L. donovani* parasites [18]. Mouse models of leishmaniasis have also demonstrated that the eosinophil responses depend on mouse strains, *Leishmania* species, and the phase of infection [11, 19, 20]. Elevated eosinophil numbers, persisting throughout the infection, have been described in susceptible mouse strains infected with *L. mexicana* and *L. amazonensis*, which correlated with enhanced tissue damage and high numbers of intracellular amastigotes [7,11]. Eosinophils employ various mechanisms to control pathogens, including oxidative bursts, phagocytosis, and the release of eosinophil extracellular traps (EETs). EETs contain histones, eosinophil cationic proteins, and cytotoxic granules such as MBP [21-22] within "degranulation sacs", which are released through a single fusion pore [23]. Furthermore, the shedding of clusters of free extracellular eosinophil granules, as well as of plasma membrane-enveloped structures containing cytoplasmic granules, has been reported. After membrane lysis, the granules are released into the extracellular space, where they function as free

granules [24].

Our findings demonstrate that eosinophils phagocytize *L. mexicana* promastigotes *in vitro* (Supplementary 1), which is accompanied by various forms of granule exocytosis that inflict damage to the parasite, as confirmed by electron microscopy and Giemsa staining. The contact between the parasites and eosinophils induced the release of free granules and the extrusion of membrane bound “degranulation sacs" containing numerous granules, which are released near the parasites, leading to damage (Supplementary 2). This phenomenon resembles a piecemeal degranulation process [23, 24]. Furthermore, eosinophils released EETs containing MBP in response to *L. mexicana* parasites. Our data align with previous reports of eosinophils attaching to and phagocytizing *Leishmania mexicana amazonensis* parasites, resulting in oxidative bursts and the release of extracellular traps containing membrane-bound clusters of granules, as well es free granules, both of which potentially contribute to parasite elimination [25-27]. Although our observations were conducted *in vitro,* this phenomenon may be expected to occur in infected tissues, thus contributing to tissue damage [28, 29].

Eosinophil cytokines modulate the immune response, but their involvement in defining the virulence of cutaneous leishmaniasis is not well understood. Leishmaniasis, a disease with divergent outcomes based on the balance of pro- and anti-inflammatory cytokines, is regulated by cytokine-producing cells. Th1 cytokines support parasite control, while a Th2 response is associated with susceptibility [6, 16]. Given that the immune response in DCL patients cannot effectively control the extensive spread of parasites, we were interested in examining whether eosinophil responses to *L. mexicana* differ between DCL and

LCL patients, potentially favoring disease progression.

Our data now show that eosinophils from DCL patients secrete significantly higher levels of IL-6, IL-8 and IL-13 in response to *L. mexicana* promastigotes, compared to eosinophils from LCL patients. This differential response between DCL and LCL eosinophils, may be related to the genetic background of patients, as previous studies have reported differential expression of inflammatory genes in NK cells from DCL and LCL patients following TLR stimulation with LPG, with LCL NK cells expressing more genes associated to inflammatory responses compared to DCL NK cells [30].

Previous studies have reported the presence of IL-6 during the active phases of infection by several *Leishmania* species [31- 33]. IL-6 along with IL-1 and IL-23 regulates Th17 cell differentiation [33]. The Th17 response promotes the chemotaxis of neutrophils through the induction of IL-8 on fibroblasts, epithelial, and endothelial cells [34]. In experimental cutaneous leishmaniasis, the Th17 response has been associated with an increase in pathological conditions, primarily due to the neutrophil-mediated promotion of Th17 response [35].

Early recruitment of neutrophils to the infection site mediated by IL-8 also leads to their activation and enhanced phagocytosis of the parasite. Neutrophils not only harbor the parasite, shielding it from the deleterious effects of complement, but they have also been described to aid in parasite spread through “silent” phagocytosis that occurs after apoptotic cells containing the parasites are phagocytosed by nonactivated macrophages (“Trojan horse” strategy) [36-38]. Our results of enhanced production of this chemokine are in line with the literature, where infections by *L. infantum,* and *L. major* have all been shown shown to

enhance the production of IL-8 [39-41]. Additionally, aside from disease modulation resulting from enhanced IL-8 production, polymorphisms in IL-8 (−251A/T) or IL-6 (174G/C) genes should also be considered in future studies, as they have been shown to influence the outcome of mucosal leishmaniasis [42-44]. This aspect remains to be analyzed in cutaneous leishmaniasis caused by *L. mexicana.*

Our finding of elevated production of IL-13 by eosinophils of DCL patients is another factor that might contribute to enhanced disease severity. Il-13 activates eosinophils and promotes the development of alternatively activated macrophages, which have been shown to promote disease progression in cutaneous Leishmaniasis [16, 33, 36]. This cytokine is functionally redundant with IL-4 and inhibits the production of inflammatory cytokines, thereby interfering with resistance to *Leishmania* [45]. Furthermore, IL-13 can promote the isotype switching of B cells to produce IgE and IgG4 in humans [46]. Our observations reveal that both DCL and LCL patients have elevated anti-*Leishmania* IgE and IgG antibodies. Although no differences were observed in IgE production between both clinical forms, it is noteworthy that both patient groups tested positive for IgE, contrasting with other reports of IgE in *L. amazonensis* infections, where only 40% of patients were positive for this antibody isotype [47]. However, we found that DCL patients had significantly higher levels of IgG, compared to LCL patients. This is noteworthy, as higher amounts of IgG in leishmaniasis have been associated with susceptibility, as IgG induces the production of regulatory IL-10 in monocytes, contributing to the inhibition of cellular responses in chronic DCL patients [48].

In summary, these three cytokines have been associated with disease susceptibility, and our data align with the literature, showing their enhanced

production by eosinophils from DCL patients under *in vitro* conditions. It is possible the higher eosinophil numbers in the infiltrated inflammatory areas of DCL patients contribute to the production of these cytokines, further exacerbating disease severity. However, this hypothesis remains to be investigated in the context of cutaneous leishmaniasis caused by *L. mexicana.*

Our results now shed new light on the apparent dual role of eosinophils in leishmaniasis. They can inflict crucial damage to the parasite, and produce cytokines such as IL-6, IL-8 and IL-13 upon contact with the parasite, which are associated with disease severity and pathological conditions.

Conclusions

Eosinophils employ diverse effector mechanisms when co-incubated with *Leishmania mexicana,* including the exocytosis of granules onto the parasite, the release of membrane bound "degranulation sacs", and the formation of EETs, all of which contribute to parasite damage. However, despite presenting eosinophilia, DCL patients exhibit a high parasite load and uncontrolled disease progression. Given that eosinophils from DCL patients also release significantly higher amounts of cytokines IL-6, IL-13, and the chemokine IL-8, compared to LCL patients, these mediators may contribute to the disease progression observed in DCL patients. Our data provide additional insights into the innate defense mechanisms exerted by eosinophils against *Leishmania mexicana*. To further clarify the biological significance of eosinophils in leishmaniasis, it would be interesting to study other effector mechanisms, including signaling pathways, signal transductions, and polymorphisms in the eosinophils of these patients, in order to enhance our understanding of eosinophils in this disease.

Conflict of Interest

We declare that this work has not been influenced by any financial, personal, or professional interest.

Author Contributions

Conceptualization: NSS, RPA, JDD, APT and IB

Formal Analysis: ICCG, JZC, JDD, HARM

Funding Acquisition: IB

Investigation GCF, NSS, ICCG, APT, JZC, VJLA, BSB, ARR, JDD, RPA and RBCS.

Methodology: NSS, APT, ARR, JDD and RPA

Project Administration: IB

Resources**:** BSB, VJLA, GCF

Supervision: IB

Visualization: GCF, NSS, ICCG, APT, JZC, VJLA, BSB and RBCS.

Writing – Original Draft Preparation: RPA and NSS

Writing – Review & Editing: ICCG, APT, JZC and IB

Funding Statement

The funders had no role in study design, data collection and analysis, decision to publish or preparation of the manuscript.

References

1. Scorza BM, Carvalho EM, Wilson ME. Cutaneous Manifestations of Human and Murine Leishmaniasis. Int J Mol Sci. 2017 Jun 18;18(6):1296.

2. Gabriel Á, Valério-Bolas A, Palma-Marques J, Mourata-Gonçalves P, Ruas P, Dias-Guerreiro T, et al. Cutaneous Leishmaniasis: The Complexity of Host's Effective Immune Response against a Polymorphic Parasitic Disease. J Immunol Res. 2019 Dec 1;2019:2603730.

3. Leishmaniases. Epidemiological report on the region of the Americas. Pan American Health Organization. No. 11 Dec 2022. Consulted January 2023. Available from: https://iris.paho.org/bitstream/handle/10665.2/56831/PAHOCDEVT220021_eng.pdf?sequence=1&isAllowed=y

4. Conceição-Silva F, Reis CSM, De Luca PM, Leite-Silva J, Santiago MA, Morrot A, et al. The Immune System Throws Its Traps: Cells and Their Extracellular Traps in Disease and Protection. Cells. 2021 Jul 26;10(8):1891.

5. Klion AD, Ackerman SJ, Bochner BS. Contributions of Eosinophils to Human Health and Disease. Annu Rev Pathol. 2020 Jan 24;15:179-209.

6. Ramirez GA, Yacoub MR, Ripa M, Mannina D, Cariddi A, Saporiti N, et al. Eosinophils from Physiology to Disease: A Comprehensive Review. Biomed Res Int. 2018 Jan 28;2018:9095275.

7. Grimaldi G Jr, Soares MJ, Moriearty PL. Tissue eosinophilia and *Leishmania mexicana mexicana* eosinophil interactions in murine cutaneous leishmaniasis. Parasite Immunol. 1984 Sep;6(5):397-408.

8. Bittencourt AL, Barral A, Costa JM, Saldanha AC, Badaró F, Barral-Netto M, et al. Diffuse cutaneous leishmaniasis with atypical aspects. Int J Dermatol. 1992 Aug;31(8):568-70.

9. Cáceres-Dittmar G, Tapia FJ, Sánchez MA, Yamamura M, Uyemura K, Modlin RL, et al. Determination of the cytokine profile in American cutaneous leishmaniasis using the polymerase chain reaction. Clin Exp Immunol. 1993 Mar;91(3):500-5.

10. Saito S, Hamada A, Watanabe N, Obata T, Katakura K, Ohtomo H. Eosinophil chemotactic activity in *Leishmania amazonensis* promastigotes. Parasitol Res. 1996;82(6):485-9.

11. de Oliveira Cardoso F, de Souza Cda S, Mendes VG, Abreu-Silva AL, Gonçalves da Costa SC, Calabrese KS. Immunopathological studies of *Leishmania amazonensis* infection in resistant and in susceptible mice. J Infect Dis. 2010 Jun 15;201(12):1933-40.

12. Watanabe Y, Hamaguchi-Tsuru E, Morimoto N, Nishio Y, Yagyu K, Konishi Y, et al. IL-5-Induced Eosinophils Suppress the Growth of *Leishmania amazonensis* In Vivo and Kill Promastigotes In Vitro in Response to Either IL-4 or IFN-gamma. DNA Cell Biol. 2004 Jul;23(7):412-8.

13. Salaiza-Suazo N, Volkow P, Tamayo R, Moll H, Gillitzer R, Pérez-Torres A, et al. Treatment of two patients with diffuse cutaneous leishmaniasis caused by *Leishmania mexicana* modifies the immunohistological profile but not the disease outcome. Trop Med Int Health. 1999 Dec;4(12):801-11.

14. Kovács I, Horváth M, Kovács T, Somogyi K, Tretter L, Geiszt M, et al. Comparison of proton channel, phagocyte oxidase, and respiratory burst levels between human eosinophil and neutrophil granulocytes. Free Radic Res. 2014 Oct;48(10):1190-9.

15. Real F, Pouchelet M, Rabinovitch M. *Leishmania* (*L.*) *amazonensis*: fusion between parasitophorous vacuoles in infected bone-marrow derived mouse macrophages. Exp Parasitol. 2008 May;119(1):15-23.

16. Ariyaratne A, Finney CAM. Eosinophils and Macrophages within the Th2-Induced Granuloma: Balancing Killing and Healing in a Tight Space. Infect Immun. 2019 Sep 19;87(10):e00127-19.

17. Lezama-Dávila CM, Isaac-Márquez AP, Padierna-Olivos J, Aguilar-Torrentera F, Chapa-Ruiz R. Immunomodulation of Chiclero's ulcer. Role of eosinophils, T cells, tumour necrosis factor and interleukin-2. Scand J Immunol. 1998 May;47(5):502-8.

18. Wijesinghe H, Gunathilaka N, Semege S, Pathirana N, Manamperi N, de Silva C, et al. Histopathology of Cutaneous Leishmaniasis Caused by *Leishmania donovani* in Sri Lanka. Biomed Res Int. 2020 May 2;2020:4926819.

19. Monteiro MC, Lima HC, Souza AA, Titus RG, Romão PR, Cunha FQ. Effect of *Lutzomyia longipalpis* salivary gland extracts on leukocyte migration induced by *Leishmania major*. Am J Trop Med Hyg. 2007 Jan;76(1):88-94.

20. Figueredo LA, de Paiva-Cavalcanti M, Almeida EL, Brandão-Filho SP, Dantas-Torres F. Clinical and hematological findings in *Leishmania braziliensis*-infected dogs from Pernambuco, Brazil. Rev Bras Parasitol Vet. 2012 Oct-Dec;21(4):418-20.

21. Williams TL, Rada B, Tandon E, Gestal MC. "NETs and EETs, a Whole Web of Mess". Microorganisms. 2020 Dec 4;8(12):1925.

22. Soragni A, Yousefi S, Stoeckle C, Soriaga AB, Sawaya MR, Kozlowski E, et al. Toxicity of eosinophil MBP is repressed by intracellular crystallization and promoted by extracellular aggregation. Mol Cell. 2015 Mar 19;57(6):1011-1021.

23. Crivellato E, Nico B, Mallardi F, Beltrami CA, Ribatti D. Piecemeal degranulation as a general secretory mechanism? Anat Rec A Discov Mol Cell Evol Biol. 2003 Sep;274(1):778-84.

24. Fettrelet T, Gigon L, Karaulov A, Yousefi S, Simon HU. The Enigma of Eosinophil Degranulation. Int J Mol Sci. 2021 Jun 30;22(13):7091. doi: 10.3390/ijms22137091.

25. Pimenta PF, Dos Santos MA, De Souza W. Fine structure and cytochemistry of the interaction between *Leishmania mexicana amazonensis* and rat neutrophils and eosinophils. J Submicrosc Cytol. 1987 Jul;19(3):387-95.

26. Ueki S, Melo RC, Ghiran I, Spencer LA, Dvorak AM, Weller PF. Eosinophil extracellular DNA trap cell death mediates lytic release of free secretion-competent eosinophil granules in humans. Blood. 2013 Mar 14;121(11):2074-83.

27. Guimarães-Costa AB, Nascimento MT, Froment GS, Soares RP, Morgado FN, Conceição-Silva F, et al. *Leishmania amazonensis* promastigotes induce and

are killed by neutrophil extracellular traps. Proc Natl Acad Sci U S A. 2009 Apr 21;106(16):6748-53.

28. Rodríguez NE, Wilson ME. Eosinophils and mast cells in leishmaniasis. Immunol Res. 2014 Aug;59(1-3):129-41.

29. Nija RJ, Sanju S, Sidharthan N, Mony U. Extracellular Trap by Blood Cells: Clinical Implications. Tissue Eng Regen Med. 2020 Apr;17(2):141-153.

30. Fernández-Figueroa EA, Imaz-Rosshandler I, Castillo-Fernández JE, Miranda-Ortíz H, Fernández-López JC, Becker I et al. (2016). Down-Regulation of TLR and JAK/STAT Pathway Genes Is Associated with Diffuse Cutaneous Leishmaniasis: A Gene Expression Analysis in NK Cells from Patients Infected with *Leishmania mexicana*. PLoS Negl Trop Dis. 2016 Mar 31; 10(3): e0004570.

31. de Lima VM, Peiro JR, de Oliveira Vasconcelos R. IL-6 and TNF-alpha production during active canine visceral leishmaniasis. Vet Immunol Immunopathol. 2007 Jan 15;115(1-2):189-93.

32. Dos Santos PL, de Oliveira FA, Santos ML, Cunha LC, Lino MT, de Oliveira MF, et al. The Severity of Visceral Leishmaniasis Correlates with Elevated Levels of Serum IL-6, IL-27 and sCD14. PLoS Negl Trop Dis. 2016 Jan 27;10(1):e0004375.

33. Maspi N, Abdoli A, Ghaffarifar F. Pro- and anti-inflammatory cytokines in cutaneous leishmaniasis: a review. Pathog Glob Health. 2016 Sep;110(6):247-260.

34. Huang N, Dong H, Luo Y, Shao B. Th17 Cells in Periodontitis and Its Regulation by A20. Front Immunol. 2021 Sep 7; 12:742925.

35. Pedraza-Zamora CP, Delgado-Domínguez J, Zamora-Chimal J, Becker I. Th17 cells and neutrophils: Close collaborators in chronic *Leishmania mexicana* infections leading to disease severity. Parasite Immunol. 2017 Apr;39(4).

36. Dayakar A, Chandrasekaran S, Kuchipudi SV, Kalangi SK. Cytokines: Key Determinants of Resistance or Disease Progression in Visceral Leishmaniasis: Opportunities for Novel Diagnostics and Immunotherapy. Front Immunol. 2019 Apr 5;10:670.

37. Beste MT, Lomakina EB, Hammer DA, Waugh RE. Immobilized IL-8 Triggers Phagocytosis and Dynamic Changes in Membrane Microtopology in Human Neutrophils. Ann Biomed Eng. 2015 Sep;43(9):2207-19.

38. Carreira, JC, da Silva AV. The Role of Neutrophils in the Interaction with *Leishmania*: Far beyond a Simple Trojan Horse?. Open J. Anim. Sci. 2021 Jun 25; 11, 399-421.

39. Badolato R, Sacks DL, Savoia D, Musso T. *Leishmania major*: infection of human monocytes induces expression of IL-8 and MCAF. Exp Parasitol. 1996 Jan;82(1):21-6.

40. Boussoffara T, Boubaker MS, Ben Ahmed M, Mokni M, Guizani I, Ben Salah A, et al. Histological and immunological differences between zoonotic cutaneous leishmaniasis due to *Leishmania major* and sporadic cutaneous leishmaniasis due to *Leishmania infantum*. Parasite. 2019;26:9.

41. Oualha R, Barhoumi M, Marzouki S, Harigua-Souiai E, Ben Ahmed M, Guizani I. Infection of Human Neutrophils With *Leishmania infantum* or *Leishmania major* Strains Triggers Activation and Differential Cytokines Release. Front Cell Infect Microbiol. 2019 May 10;9:153.

42. Hajilooi M, Abasi M, Bazmani A, Ahmadi A, Matini M, Solgi G, et al. Evaluation of interleukin-8 -251 t/a polymorphisms in visceral leishmaniasis. J Res Health Sci. 2015 Winter;15(1):59-61.

43. Castellucci L, Menezes E, Oliveira J, Magalhaes A, Guimaraes LH, Lessa M, et al. IL6 -174 G/C promoter polymorphism influences susceptibility to mucosal but not localized cutaneous leishmaniasis in Brazil. J Infect Dis. 2006 Aug 15;194(4):519-27.

44. Cardoso T, Bezerra C, Medina LS, Ramasawmy R, Scheriefer A, Bacellar O, et al. *Leishmania braziliensis* isolated from disseminated leishmaniasis patients downmodulate neutrophil function. Parasite Immunol. 2019 May;41(5):e12620.

45. Zaatar MT, Simaan Y, Karam MC. Exogenous IL-13 exacerbates *Leishmania major* infection and abrogates acquired immunity to re-infection. Parasitol Res 2022 Jul;121(7):2009-2017.

46. Le Floc'h A, Allinne J, Nagashima K, Scott G, Birchard D, Asrat S, et al. Dual blockade of IL-4 and IL-13 with dupilumab, an IL-4Rα antibody, is required to broadly inhibit type 2 inflammation. Allergy. 2020 May;75(5):1188-1204.

47. Sousa-Atta ML, Salamé GS, D'Oliveira A Jr, Almeida RP, Atta AM, Carvalho EM. Immunoglobulin E antileishmanial antibody response in cutaneous leishmaniasis. Clin Diagn Lab Immunol. 2002 Jan;9(1):101-4.

48. Buxbaum LU. *Leishmania mexicana* infection induces IgG to parasite surface glycoinositol phospholipids that can induce IL-10 in mice and humans. PLoS Negl Trop Dis. 2013 May 9;7(5):e2224.

Figure legends

Figure 1. Eosinophils in Peripheral Blood of Patients with Cutaneous Leishmaniasis.

A. Percentage of blood eosinophils from patients with LCL (green circles, n = 30) and DCL (red squares, n = 4). B. Blood eosinophils and disease duration. The horizontal line represents the mean, and each dot represents a patient (female or male). Bars represent the mean ± SEM. ns: non-significant.

Figure 2. Eosinophils in Lesions of Patients with Cutaneous Leishmaniasis.

Eosinophils in the tissue were quantitated and expressed as eosinophils/mm^2. The bar graph shows the mean, and each dot represents a patient. Representative H&E staining of biopsies taken from LCL patients (green circles, n=35) and DCL patients (red squares, n=5) are shown. Black arrows indicate *Leishmania* amastigotes and blue arrows indicate eosinophils. Scale bar = 20 µm. Bars represent the mean ± SEM, and asterisks represent statistically significant differences ($P < 0.05$).

Figure 3. Anti-*Leishmania* IgE and IgG in Sera from Patients with Cutaneous Leishmaniasis.

A. IgE levels. B. IgG levels. Each symbol represents a patient, and the horizontal line represents the mean of each group. LCL (n=8) and DCL (n=8) patients, healthy controls (n=8). *All symbols represent statistically significant differences ($P < 0.05$).

Figure 4. Cytokine Production and Oxidative Burst were Analyzed in Eosinophils from Patients with LCL and DCL.

Eosinophils from LCL and DCL patients and from healthy controls were incubated with *L. mexicana* promastigotes. A. IL-6 production. B. IL-8 production. C. IL-13 production. D. ROS production measured by luminol reaction (in mV). Bars represent the mean ± SEM (n= 7 for each group of patients and healthy control subjects). All symbols represent statistically significant differences ($P < 0.05$).

Figure 5. Phagocytosis and Lysis of *Leishmania mexicana* by Eosinophils.

A. Purified eosinophils in the absence of parasites. B. Eosinophils incubated with *L. mexicana* promastigotes (Giemsa stained). Close contact between parasites and cells leads to abundant degranulation (green arrow) and damaged parasites (black arrows). C. *Leishmania* promastigotes stained with CFDA. D. Eosinophils co-incubated with *Leishmania* promastigotes show phagocytosed parasite (black arrow). Scale bar = 20 µm. E. Percentage of eosinophil degranulation with or without *L. mexicana* (n=3). Bars represent the mean ± SD, and the asterisk represents statistically significant differences ($P < 0.05$).

Figure 6. Release of Cytoplasmic “Degranulation Sacs” by Eosinophils Co-incubated with Parasites.

A. Eosinophil (green arrow) and release of cytoplasmic “degranulation sacs". B. Degranulation sacs (black arrows) and parasites (pink arrow). C and D: Ultramicroscopic images showing released specific or secondary granules (g) or within cytoplasmic pockets limited by an intact membrane (black arrows). These “degranulation sacs” were in close apposition to distorted *L. mexicana* (Lm). Parasites, identified by their submembranous microtubule cytoskeleton (arrowheads) show signs of damage as evidenced by osmiophilic areas (white asterisk), nuclear fragmentation (n), and a clear or aqueous cytoplasm denoting an extensive cytoplasmic vacuolization. (D) Some eosinophil specific granules still retain the characteristic electron-dense central crystalline core, surrounded by a clearer peripheral matrix (red arrows). Light microscopy scale bar = 20 µm.

Figure 7. Extracellular Eosinophil Traps Induced by Co-incubation with *L. mexicana*.

A) Extracellular traps (black asterisk) containing MBP-positive granules (black arrow) and parasites within the nets (red arrow). B) Purified eosinophils show their nuclei stained blue with DAPI (green arrow). No nets are formed in the absence of *Leishmania.* C) Eosinophils co-incubated with *L. mexicana* promastigotes show the formation of extracellular DNA traps (white arrow). D) The same image (C) was analyzed with light and fluorescence microscopy, showing the parasites (red arrow) in contact with nets (white arrow). Scale bar = 20 µm.

Supplementary 1. Phagocytosed Parasites by Eosinophils.

A) Parasites stained with CFDA (green color) phagocytosed by eosinophils are observed inside these cells. Black arrows show phagocytized parasites. B) Remains of degraded parasites (black arrows) are observed already inside the eosinophils (green color granules).

Supplementary 2. *Leismania mexinana* Damaged by Eosinophil Contact.

A) Normal shape and size of a viable *Leishmania* promastigote. B) Morphological changes in parasite size, damage to membranes, formation of small vacuoles within the parasite, and loss of flagellum were observed in parasites co-incubated with eosinophils in a 1:10 ratio for 1hour. Black arrows show damaged parasites.

Figure 1

Click here to access/download;Figure;Figure 1.tiff

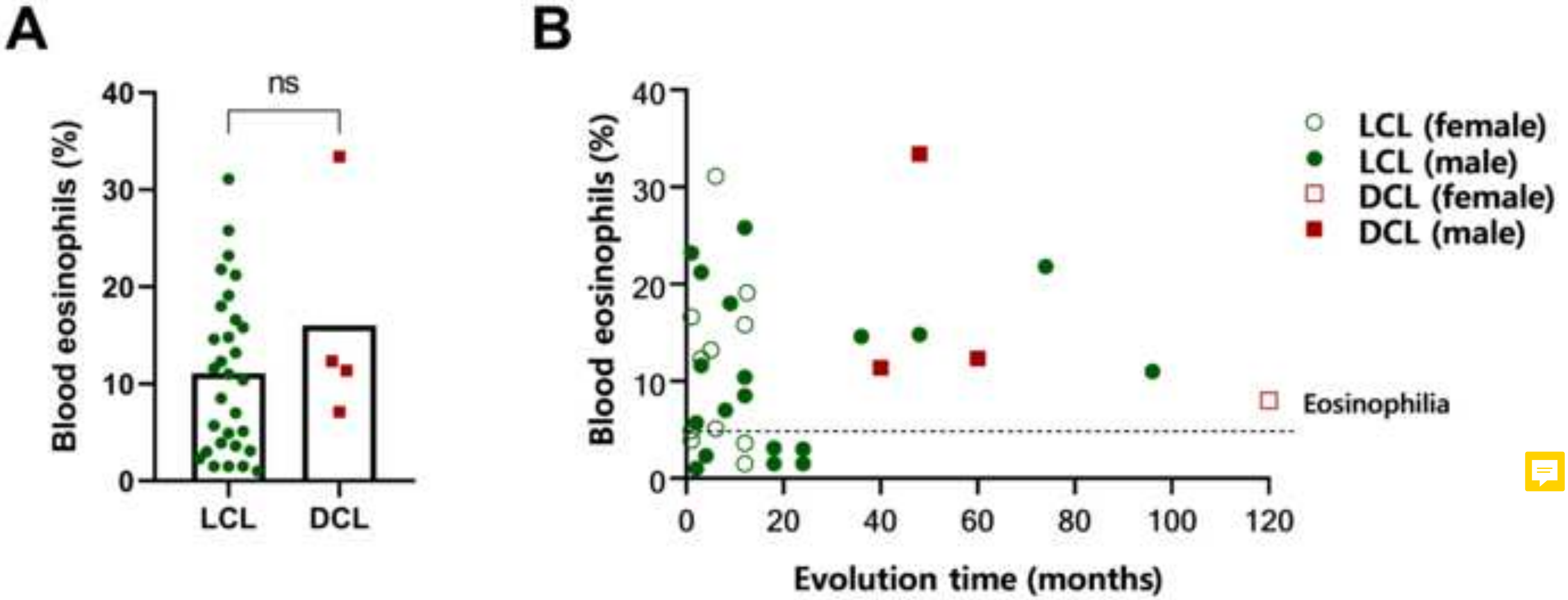

Figure 2

Click here to access/download;Figure;Figure 2.tiff

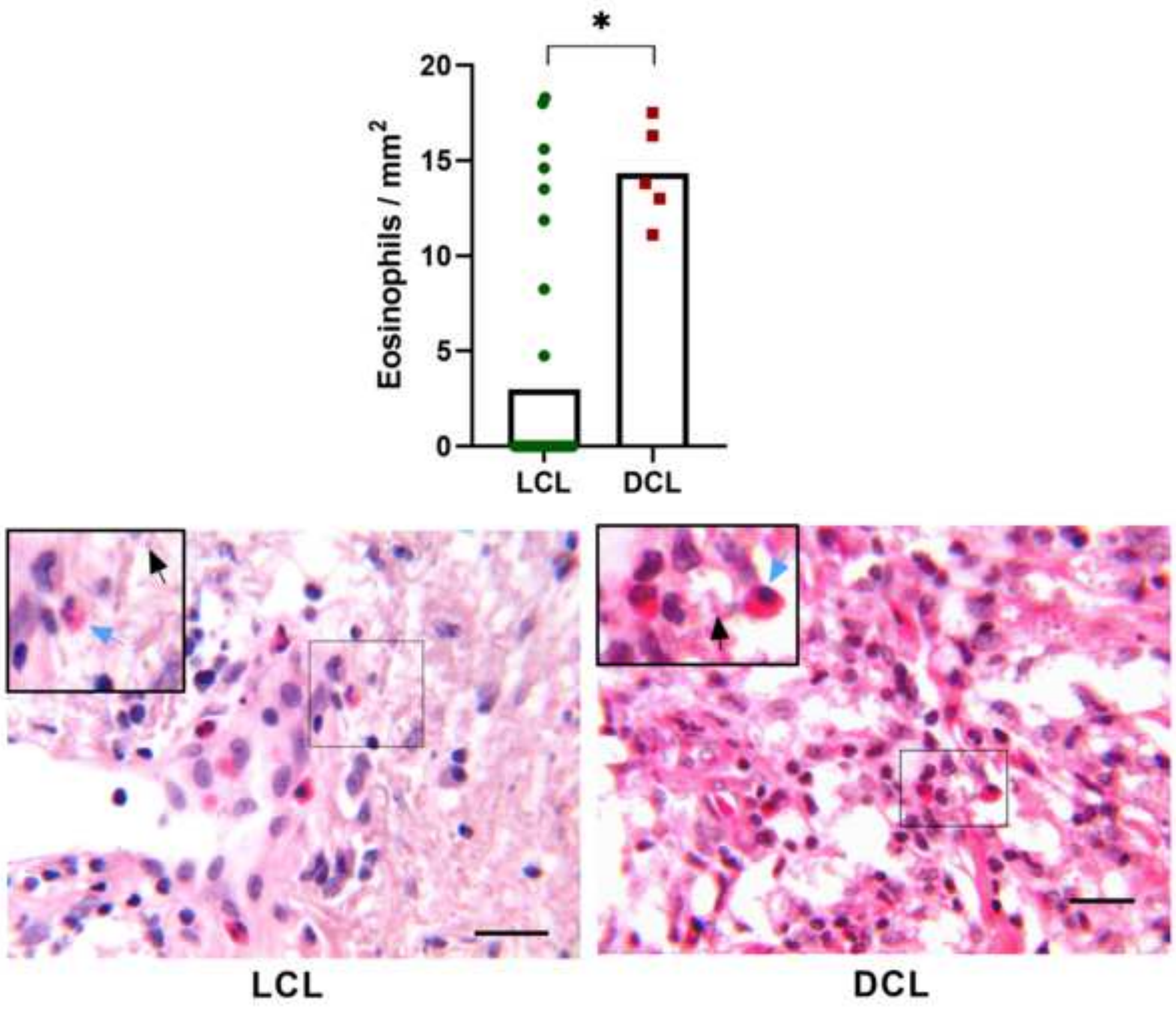

Figure 3

Click here to access/download;Figure;Figure 3.tiff

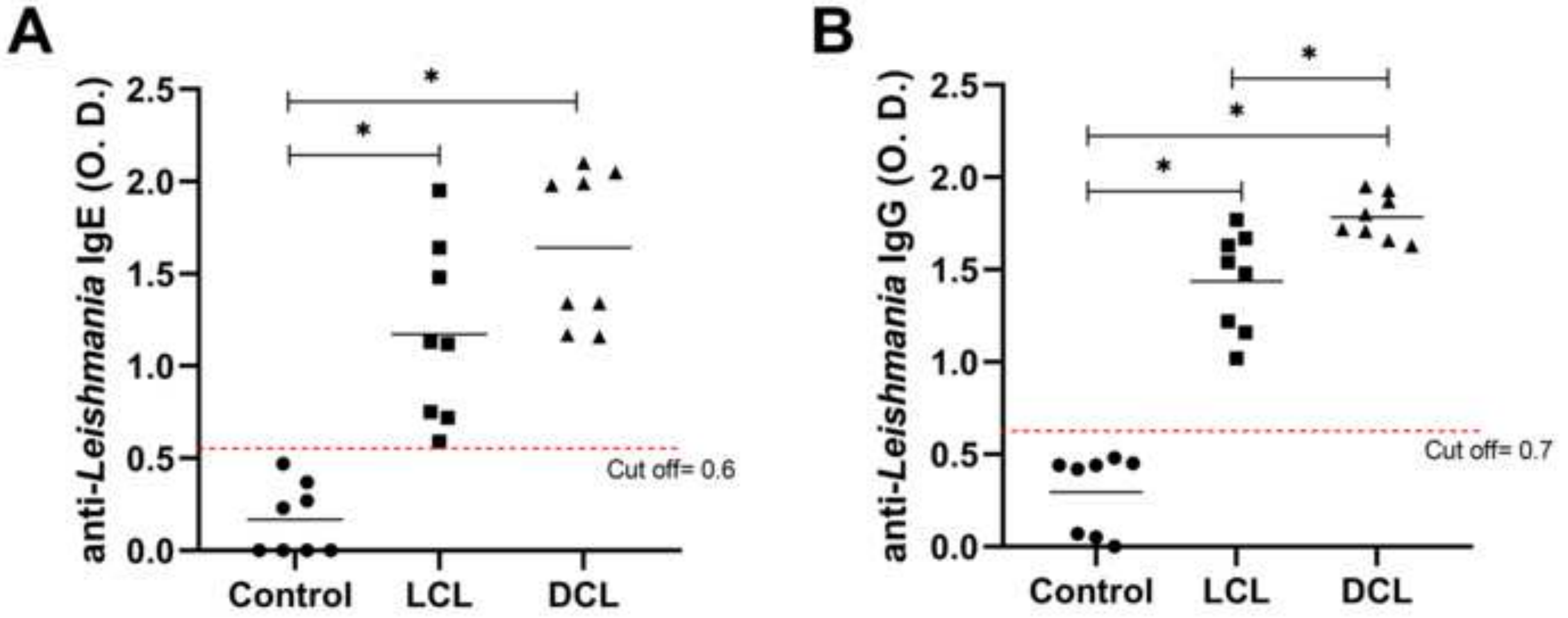

Figure 4

Click here to access/download;Figure;Figure 4.tiff

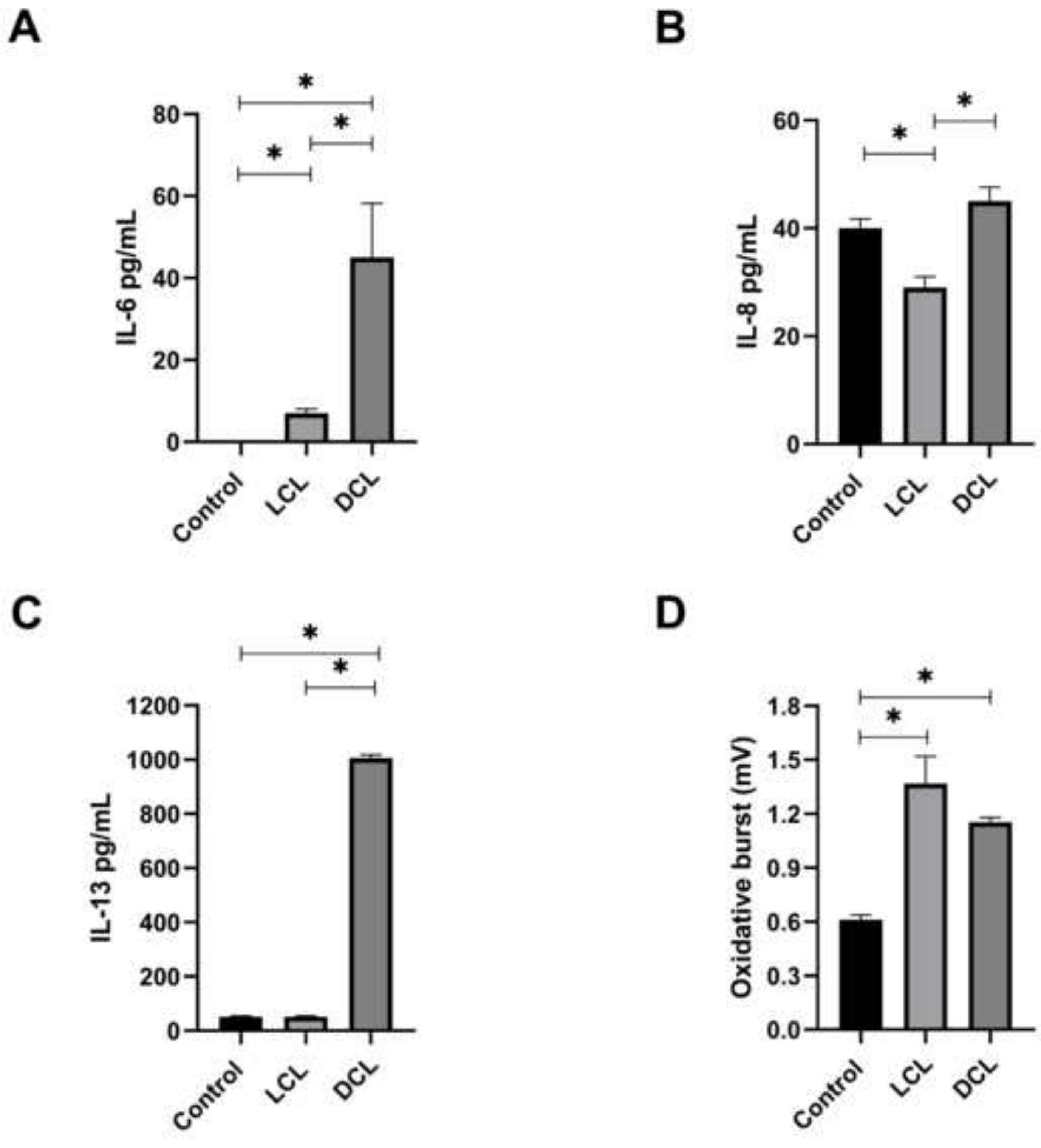

Figure 5

Click here to access/download;Figure;Figure 5.tiff

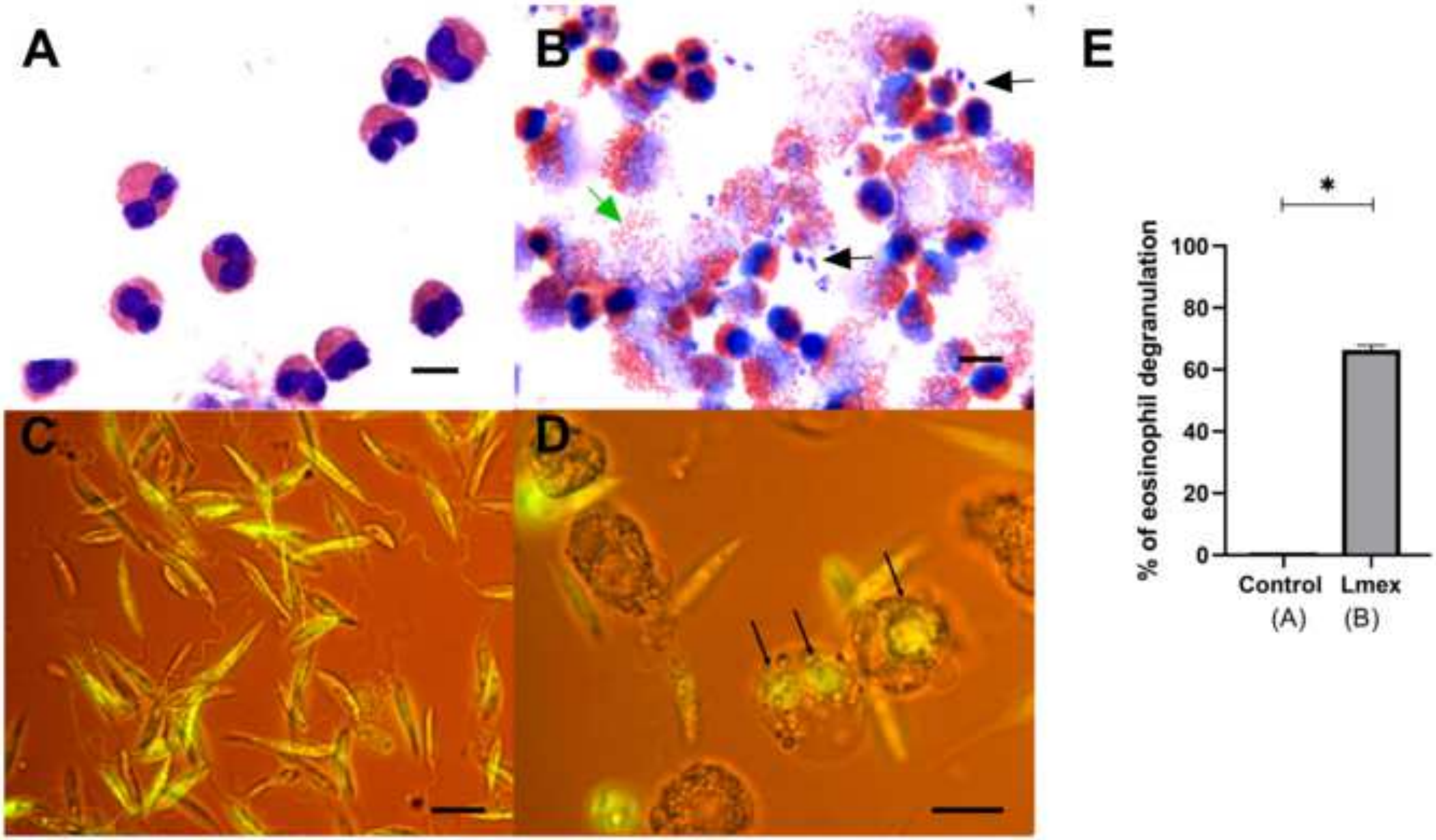

Figure 6

Click here to access/download;Figure;Figure 6.tiff

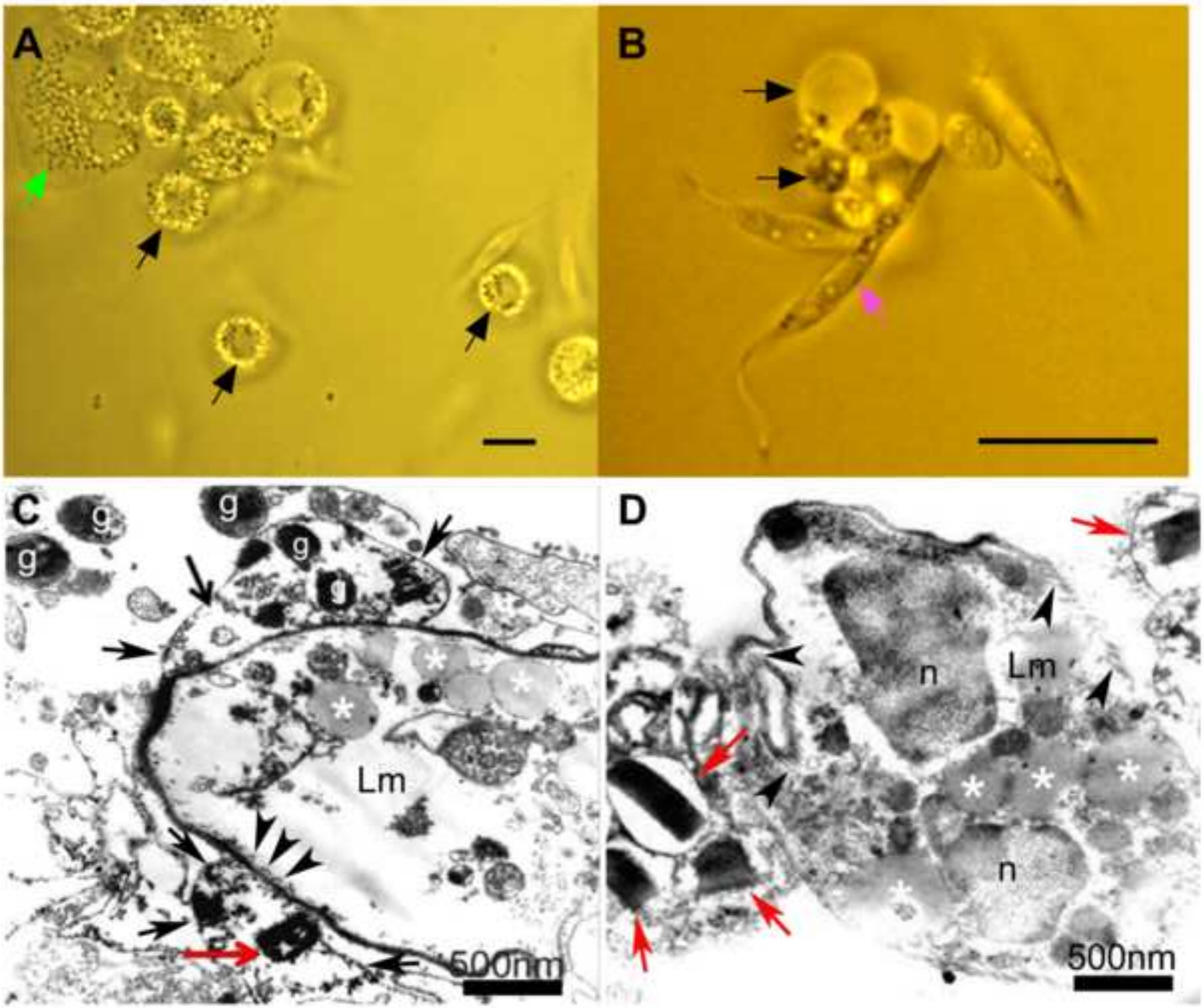

Figure 7

Click here to access/download;Figure;Figure 7.tiff

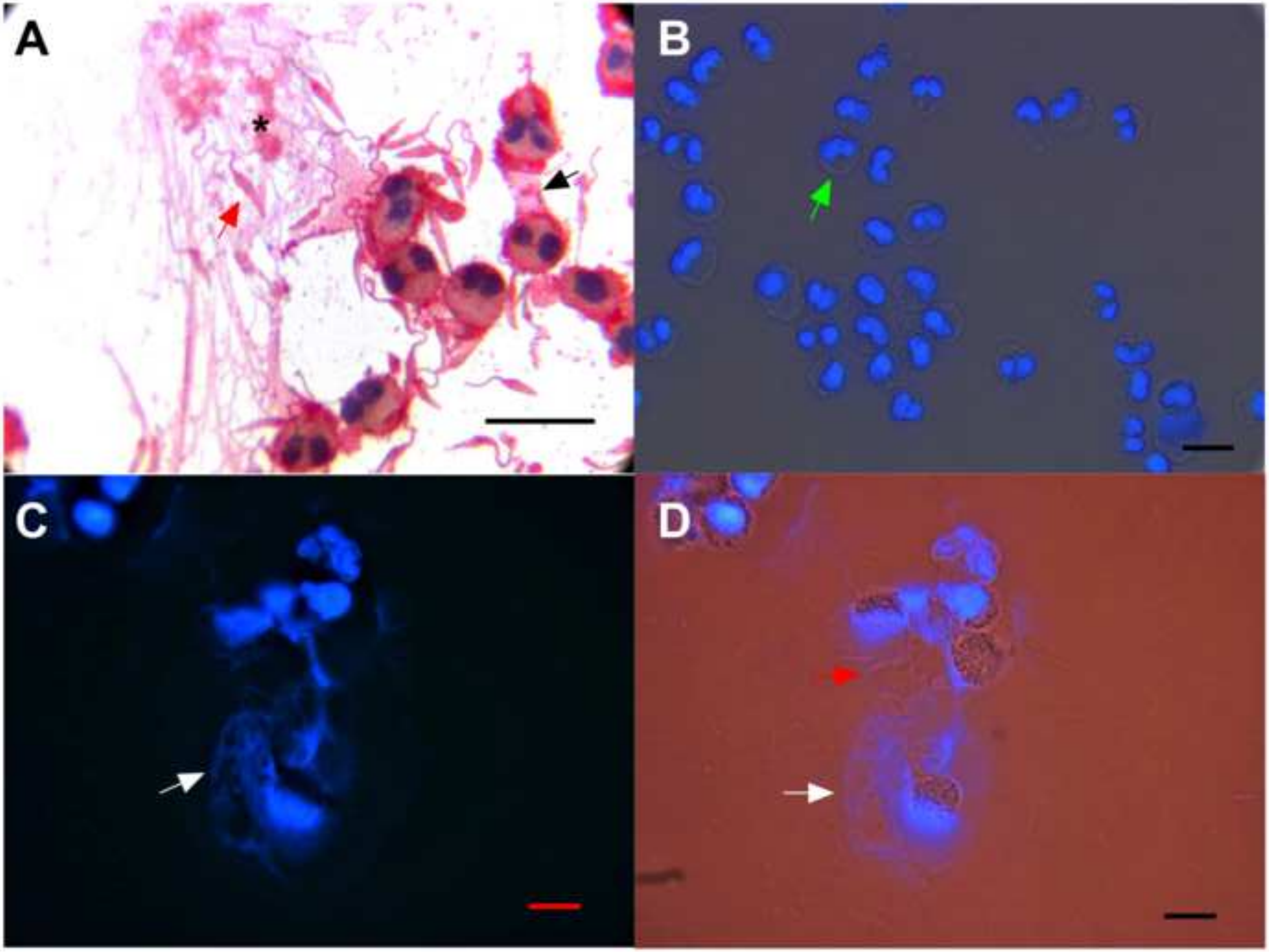

Supplementary figure 1

Click here to access/download
Supporting Information
Suplementary 1.jpg

Supplementary figure 2

Click here to access/download
Supporting Information
Suplementary 2.jpg

Eosinophils of patients with localized and diffuse cutaneous leishmaniasis: differential response to *Leishmania mexicana*, with insights into mechanisms of damage inflicted upon the parasites by eosinophils

Norma Salaiza-Suazo[1], Roxana Porcel-Aranibar[1], Isabel Cristina Cañeda-Guzmán[1], Adriana Ruiz-Remigio[1], Jaime Zamora-Chimal[1], José Delgado-Domínguez[1], Rocely Cervantes-Sarabia[1], Georgina Carrada-Figueroa[2], Baldomero Sánchez-Barragán[3], Victor Javier Leal-Ascencio[4], Armando Pérez-Torres[5], Héctor A. Rodríguez-Martínez[1], Ingeborg Becker[1*]

[1]Unidad de Investigación en Medicina Experimental, Facultad de Medicina, Universidad Nacional Autónoma de México, Ciudad de México 04510, México.

[2]División Académica de Ciencias de la Salud, Universidad Juárez Autónoma de Tabasco (UJAT), Tabasco, México

[3]Escuela de Medicina, Universidad Juárez Autónoma de Tabasco (UJAT), Tabasco, México

[4] Hospital Regional de Alta Especialidad Dr. Juan Graham, Secretaría de Salud del Estado de Tabasco, Villahermosa, Tabasco, México

[5] Departamento de Biología Celular y Tisular, Facultad de Medicina, Universidad Nacional Autónoma de México, Ciudad de México, México

*Corresponding author

Ingeborg Becker

Tel. -52-55-56232674

e-mail: becker@unam.mx

Abstract

Eosinophils are mainly associated with parasitic infections and allergic manifestations. They produce many biologically active substances, that contribute to the destruction of pathogens through the degranulation of microbicidal components and inflammatory tissue effects. In leishmaniasis, eosinophils have been found within inflammatory infiltrate with protective immunity against the parasite. We analyzed the responses of eosinophils from patients with localized (LCL) and diffuse (DCL) cutaneous leishmaniasis, as well as from healthy subjects, when exposed to *Leishmania mexicana.*

All DCL patients exhibited blood eosinophilia, along with elevated eosinophil counts in non-ulcerated nodules. In contrast, only LCL patients with prolonged disease progression showed eosinophils in their blood and cutaneous ulcers. Eosinophils from DCL patients secreted significantly higher levels of IL-6, IL-8, and IL-13, compared to eosinophils from LCL patients. Additionally, DCL patients displayed higher serum levels of anti-*Leishmania* IgG antibodies. We also demonstrated that eosinophils from both LCL and DCL patients responded to *L. mexicana* promastigotes with a robust oxidative burst, which was equally intense in both patient groups and significantly higher than in healthy subjects.

Coincubation of eosinophils (from donors with eosinophilia) with *L. mexicana* promastigotes *in vitro* revealed various mechanisms of parasite damage associated with different patterns of granule exocytosis: 1) localized degranulation on the parasite surface, 2) the release of cytoplasmic membrane-bound “degranulation sacs” containing granules, 3) release of eosinophil extracellular traps containing DNA and granules with major basic protein.

In conclusion, eosinophils damage *L. mexicana* parasites through the release of granules via diverse mechanisms. However, despite DCL patients having abundant eosinophils in their blood and tissues, their apparent inability to provide protection may linked to the release of cytokines and chemokines that promote a Th2 immune response and disease progression in these patients.

Introduction

In humans, *Leishmania mexicana* can cause localized cutaneous leishmaniasis (LCL) and diffuse cutaneous leishmaniasis (DCL). The most common clinical form of the disease occurring in over 90% of the cases is LCL. It is characterized by ulcers at the sites of parasite inoculation, accompanied by a robust cellular immune response and a scarce number of parasites within the lesions. Patients with LCL generally experience a relatively benign clinical course [1]. Less than 1% progress to DCL, which is characterized by multiple non-ulcerated nodules containing heavily parasitized macrophages that cover large areas of the body. These patients exhibit an ineffective cell-mediated immune response against *Leishmania* parasites, leading to uncontrolled and potentially fatal progression [2]. In Mexico, 500-1000 new cases of cutaneous leishmaniasis are diagnosed every year [3].

In the early stages of infection, the inflammatory infiltrate includes neutrophils, macrophages, and eosinophils. Under normal conditions, eosinophils constitute only 1–5% of circulating leukocytes, although they can also be found in tissues as resident cells [4].

Eosinophils are well equipped to combat pathogens, such as helminths, and their numbers increase during infection [5]. Eosinophils play key roles in immune

modulation, tissue remodeling, and repair, primarily through the secretion and degranulation of inflammatory mediators, including cytokines and chemokines. However, their effector mechanisms can also inadvertently damage bystander cells and tissues [5, 6].

During the acute phase of leishmaniasis, eosinophils can be present in skin lesions, often alongside mast cells. Furthermore, a close association between eosinophils and parasitized macrophages has been reported in chronic lesions, suggesting that eosinophils may contribute to parasite destruction through cooperation with macrophages [7]. Tissue eosinophilia has been observed in DCL patients, with evidence of parasitized and lysed eosinophils and dispersion of their granules near parasitized macrophages [8]. Additionally, both in the mouse model of leishmaniasis and in humans with chronic phases of the disease, eosinophils are frequently found in granulomas [9, 10, 11]. One of the mechanisms through which eosinophils assist in controlling the parasite is the production of hydrogen peroxide, which has a detrimental effect on *Leishmania amazonensis* [12]. However, the precise physiological role of eosinophils in leishmaniasis remains incompletely understood.

In this study, we conducted a comparative analysis of the *in vitro* response of eosinophils isolated from LCL and DCL patients, following their co-incubation with *L. mexicana.* Our analysis included the measurement of IL-6, IL8 and IL-13 production, as well as assessment of their oxidative burst.

Additionally, we co-incubated eosinophils from non-*Leishmania* eosinophilic subjects with *L. mexicana* promastigotes and analyzed their defense mechanisms against the parasites. Our findings reveal that eosinophils employ various defense

mechanisms when confronted with *L. Mexicana.* These mechanisms include phagocytosis, contact degranulation, the release of granules directly onto the parasite, expulsion of “degranulation sacs” containing granules surrounded by a membrane, which are released into the immediate vicinity of the parasites, and the release of eosinophil extracellular traps (EETs) containing DNA and granules rich in major basic protein (MBP). All of these mechanisms result in irreversible damage to the parasite. Our data provide new insights into the innate defense mechanisms exerted by eosinophils against *L mexicana*, as well as differences in the eosinophil response between LCL and DCL patients.

Materials and methods

Ethics Statement

The study received approval from the Ethics and Research Committees of the Facultad de Medicina, UNAM (Universidad Nacional Autónoma de México) with reference FM/DI/088/2017. We adhered strictly to the guidelines established by the Ministry of Health in Mexico. All participants and healthy donors (controls), were informed and provided written consent to participate in the study.

Patients

The number of participants varied across different study sections and these numbers are specified in each respective section. Patients diagnosed with LCL and DCL were residents of the State of Tabasco, an endemic area for *L. mexicana* in southeastern Mexico. All patients received anti-*Leishmania* treatment with pentavalent antimonials, as mandated by National Health Authorities. LCL patients

presented active lesions with relatively few parasites. In contrast, DCL patients exhibited multiple nodular lesions characterized by heavily parasitized macrophages. The severity of DCL cases ranged from patients with nodules confined to the upper body to those with nodules affecting the entire body, limbs and oral and nasal mucosae. *Leishmania* parasites were detected in smears created from biopsy punches taken from the lesions, stained using Giemsa and subjected to histopathological analysis with hematoxylin and eosin (H&E) staining. For *in vitro* experiments, eosinophils from individuals with eosinophilia (n=8) of unknown origin who sought care at the allergy ward in the Hospital General de Mexico, were utilized. Only individuals with eosinophilia exceeding 6% were included in this study.

Peripheral blood eosinophils were quantified via flow cytometry, using FCS and SSC parameters, in patients with LCL (n=30) and DCL (n=4). Punch skin biopsies (Stiefel Laboratories, Inc., Coral Gables, FL, USA) were obtained from the lesions of LCL (n= 35) and DCL patients (n= 5), using 2% xylocaine as a local anesthesia. The biopsy specimens were fixed in 10% formalin and embedded in paraffin (Sigma-Aldrich, St. Louis, Mo, USA), cut into 4 µm thick sections, and stained with H&E, to evaluate eosinophil infiltration. Eosinophils were counted in eight images of each tissue, with a final area corresponding to 1 mm^2, using an Axio imager M1 microscope equipped with an MRc5 digital camera and Axiovision 4.8 software (Carl Zeiss, Industrielle Messtechnik GmbH, Oberkochen Germany).

Detection of IgE and IgG in Human Serum Against Leishmania mexicana Antigens

We determined IgE and IgG levels against *L. mexicana* antigens through ELISA in LCL (n=8) and DCL patients (n=8), as well as in healthy controls (n=8). Each sample was analyzed in triplicate. Briefly, 96-well EIA/RIA plates (Corning, NY, USA) were coated with 0.6 µg/100 µl of lysed promastigotes in each well and incubated for 1 hour. The solution was removed and the plates were blocked with 200 µl of 0.5% casein for 1 hour. After blocking, 100 µl of diluted (1:5) sera from leishmaniasis patients or healthy donors were added and incubated overnight at 4ºC. The plates were washed with PBS-Tween. For IgE detection, 100 µl of rabbit anti-human IgE (Dako, Agilent Technologies, Sta Clara, Ca, USA) (1:500) was added and incubated for 1 hour, followed by washing and the addition of 100 µl of biotin goat anti-rabbit IgG (1:8000) (Thermo Fisher Scientific, Waltham, Ma, USA) for 30 minutes. After washing, 100 µl of peroxidase-conjugated streptavidin (Zymed) (1:2500) was added and incubated for 30 min. For IgG detection, 100 µl of peroxidase-conjugated goat anti-human IgG (Zymed) antibody (1:4000) was added to the plates and incubated for 1 hour. Following washing,100 µl of TMB (Tetramethylbenzidine) peroxidase substrate (Becton Dickinson, NJ, USA) was added for 20 minutes. The reaction was halted with 100 µl of 1 M phosphoric acid. Absorbance was measured at 450 nm using an ELISA microplate reader (BIO-TEK).

Eosinophil Isolation

Human eosinophils were obtained from peripheral blood samples of patients with LCL (n=30) and DCL (n=4). Cells were isolated through gradient centrifugation using Ficoll Hypaque (Sigma-Aldrich, St. Louis, Mo, USA). The blood was diluted

1:2 with sterile PBS pH7.4 and layered over 20 mL Ficoll-hypaque (density ρ = 1.077 g/mL) in a 50mL conical tube. Centrifugation was performed at 600×g for 30 minutes at 20 °C using a swinging bucket rotor. The subsequent steps involved removing and discarding the plasma. Mononuclear cells were located at the plasma:Ficoll interface, and granulocytes and erythrocytes were in the pellet. The pellet was resuspended in 1× Red Blood Cell Lysis Solution, and the complete 50 mL conical tube was filled with lysis solution. Incubation occurred for 10 minutes on ice, followed by centrifugation at 300×g for 10 minutes at 20 °C. The supernatant was carefully removed entirely. Cells were washed cells by adding 50 mL of buffer, and the cell number was determined. The cell suspension was centrifuged at 300×g for 10 minutes. The supernatant was aspirated completely, and the cells were resuspended in 50 µl of buffer per $5x10^7$ cells. To this suspension, 50 µl of anti-CD16 conjugated microbeads (Miltenyi Biotec, Bergisch-Gladbach, Germany) were added per $5x10^7$ cells, and incubation occurred for 30 minutes at 4-8 °C. Cells were washed by adding 1-2 mL of buffer per 10^7 cells, followed by centrifugation at 300×g for 10 minutes. The supernatant was discarded, and the cells were resuspended to a final volume of up to 10^8 cells in 500 µL of buffer. The suspension was then placed in a purification column, which had been previously washed with buffer and positioned in the magnetic field of a MACS separator. Unlabeled cells, including eosinophils, were obtained by depletion of magnetically labeled cells. The total effluent containing the unlabeled cell fraction harbored the enriched eosinophil fraction. The purity and viability of eosinophils were >99%, as determined by Giemsa staining and by Trypan blue exclusion, respectively.

Leishmania mexicana Promastigotes Culture

L. mexicana promastigotes were cultured in blood agar (NNN medium) overlaid with Schneider's *Drosophila* medium (Life Technologies), supplemented with 10% heat inactivated FBS (Biowest, Riverside, MO, USA) and 1% antibiotics (penicillin/streptomycin (Gibco, Life Technologies) at 28°C. Parasites were subcultured every 3-4 days and grown to a density of 1 x 10^7/ml.

Cytokine Measurement

The impact of *L. mexicana* on the production of IL-6, IL-8, and IL-13 by eosinophils from LCL (n=7) and DCL (n=7) patients, as well as healthy controls (n=7) was analyzed as follows: 1 x 10^6 cells were incubated for 18 hours at 37 °C and 5% CO_2 with 10x10^6 promastigotes in 1 ml of RPMI-1640 medium, supplemented with 10% heat-inactivated FBS. The cell-free supernatants from the cultures were collected and the concentrations of the cytokines were determined using human IL-6 and IL-13 Quantikine ELISA Kits (R&D Systems Minneapolis, MN, USA). IL-8 was analyzed by a standard sandwich ELISA. Briefly, 96-well flat-bottom microtiter plates (Costar, Corning, NY, USA) were coated with 1 µg/ml unconjugated anti-cytokine capture antibodies (Purified mouse anti-human IL-8 monoclonal antibody, Clone G265-5, PharMingen) diluted in 0.1 M Na2HPO4 (pH 9) and incubated overnight at 4°C. The plates were blocked with PBS (pH 7.4) supplemented with 0.5% casein dissolved in 0.1 N NaOH. Cell-free supernatants and recombinant hIL-8 standard (PharMingen) were incubated in RPMI-1640 medium supplemented with 10% FBS overnight at 4 °C. Bound human IL-8 was detected using 0.5 µg/ml

biotin-labeled detection antibodies (Biotinylated mouse anti-human IL-8 monoclonal antibody, Clone G265-8, PharMingen) diluted in 1% BSA with 0.05% Tween 20 and incubated for 1 hour at room temperature. The plate was developed using AP-Streptavidin Conjugate (Life TechnologiesTM) and phosphatase substrate (0.005 mg/mL, Sigma-Aldrich). Absorbance was read at 405 nm and the IL-8 concentration of each sample was calculated by regression analysis based on a standard curve. The detection limit of this assay ranged from 15.6 to 1000 pg/ml. The cut-off point is determined from the lower detection limit, values below this limit considered negative. Unstimulated eosinophils were included in all cytokine production determinations; however, they were not included in the plot as they were below the cut-off point.

Oxidative Burst

The oxidative burst was measured by chemiluminescence immunoassay with eosinophils obtained from the same group of patients and controls as in the cytokine analysis. Briefly, $1x10^6$ eosinophils were incubated with $10x10^6$ promastigotes in 80 µl of RPMI 1640 medium, 210 µl of luminol (Sigma-Aldrich, St. Louis, Mo, USA) at a concentration of 1 mg/ml, and 60 µl AB+ serum-opsonized zymosan (12.5 mg/ml). Chemiluminescence was analyzed for 30 minutes (Luminoskan Labsystem, Finland) at 37 °C with a 550 nm filter [13, 14]. The values obtained were expressed in mVolt.

Interaction of Leishmania Promastigotes and Blood Eosinophils from Healthy Controls

The interaction between *L. mexicana* and eosinophils was analyzed using 5-carboxyfluorescein diacetate (CFDA) staining. *Leishmania* promastigotes (10x10^6) were washed twice with cold RPMI and stained with 5 µM CFDA (Sigma-Aldrich, St. Louis, Mo, USA) for 10 min in RPMI at 37 ºC. The parasites were washed three times with PBS at 26 °C and incubated with 1x10^6 eosinophils for 2 hours at 37 ºC, protected from light. They were then centrifuged and resuspended in 10-20 µl of PBS and analyzed with an epifluorescent microscope [15]. Some of the smears were stained with Giemsa.

Transmission Electron Microscopy

Following careful washing with PBS, co-cultures of eosinophils (1x10^6) and promastigotes (10x10^6) were fixed in 2.5% glutaraldehyde diluted in 0.2 M cacodylate buffer, pH 7.4, for 2 hours, at 4 °C. After three washes of 10 minutes each in 0.15 M cacodylate buffer, pH 7.4 at 4 °C, the pellets of the cocultures were postfixed with 1% osmium tetroxide in 0.2 M cacodylate buffer, pH 7.4, for 30 minutes at 4 °C. Dehydration was carried out in gradually increasing concentrations of ethanol, followed by transfer to absolute toluene, using two changes of 10 minutes each. Infiltration was done with araldite 6005-toluene solution (1:1) for 24 hours at room temperature and then in pure araldite 6005 (two changes) at 60 °C for 2 hours. Specimens were embedded in pure araldite 6005 at 60 °C, for 36 h. Ultrathin sections were obtained with a diamond knife, contrasted with uranyl acetate and lead citrate, and examined using an Electron Microscope

(EM) 109 (Carl Zeiss, Germany). All reagents used were from Electron Microscopy Science, Hatfield, PA, USA.

Immunocytochemistry for Detection of Eosinophil Major Basic Protein (MBP)

For the immunocytochemical analysis, 1x 10^6 purified eosinophils were incubated in a chamber slide (Nunc Lab-Tek, Sigma-Aldrich, St. Louis, Mo, USA) for 2 hours at 37 ºC and 5% CO_2 with 10x10^6 *L. mexicana* promastigotes in 1 ml of RPMI-1640 medium supplemented with 10% heat-inactivated FBS. After incubation, they were washed with PBS and fixed with 2% paraformaldehyde (Sigma-Aldrich, St. Louis, Mo, USA). Nonspecific antigen sites were blocked with 2% bovine serum albumin, dissolved in Tris-HCl, pH 7.6. Thereafter, they were stained with mouse anti-human MBP at a dilution of 1:100 (Santa Cruz Biotechnology, Ca, USA) for 30 minutes. After washing, eosinophils were incubated with the secondary antibody, biotin goat anti-mouse, at a dilution of 1:100 (Invitrogen, Waltham, Ma, USA) for 30 minutes, washed and incubated with streptavidin AP (Alkaline Phosphatase) at a dilution of 1:100 (Thermo Fisher Scientific, Waltham, Ma, USA) for 30 minutes. After washing, they were counterstained with hematoxylin and mounted with resin. Additionally, the incubation of eosinophils with promastigotes was also stained with DAPI in separate samples (Sigma-Aldrich, St. Louis, Mo, USA) to verify the formation of DNA nets.

Statistical Analysis

Comparisons between experimental groups and controls were performed using Mann–Whitney U-test. A value of $p < 0.05$ was considered statistically significant, using Prism 8 for Windows.

Results

Eosinophils in Peripheral Blood and Lesions of LCL and DCL Patients

Blood samples were collected from 30 LCL patients (19 males and 11 females), with a mean age of 28 years and disease durations ranging from 1.5 to 18 months (16 ± 3.98 months). Four DCL patients (three males and one female) with a mean age of 37 years (range: 20 - 45 years) and disease durations ranging from 40 - 120 months were also included. Eosinophils were quantified in peripheral blood using flow cytometry. The mean percentage of eosinophils in LCL patients was 11.1 ± 1.51 (Fig. 1A), with 60% of LCL patients exhibiting eosinophilia ranging from 5% to 32% (Fig. 1B). There were no significant correlations between the number of eosinophils in peripheral blood and disease duration, age, or gender (data not shown). Notably, all female patients had a shorter disease duration compared to male patients (Fig. 1B), with females showing an average disease evolution of 6.5 ± 1.45 months, while males had an average of 21.4 ± 5.95 months.

All DCL patients exhibited blood eosinophilia, with a mean percentage of eosinophils in the blood of 16 ± 5.8%, ranging from 7% to 34% (Fig. 1A). It is noteworthy that all LCL and DCL patients with chronic infections lasting over two years had blood eosinophilia (Fig. 1B).

Eosinophils in Lesions of LCL and DCL Patients

Skin biopsies were obtained from 35 LCL patients and 5 DCL patients. When analyzing skin lesions, significant differences in eosinophil numbers were observed between both clinical forms. Eosinophils were observed in skin lesions of all 5 DCL patients, with a mean of 14 ± 1.15 eosinophils/mm^2 (ranging from 11 to18). In contrast, only 22% of LCL patients had eosinophils in their tissues, with a mean value of 3 ± 1 eosinophils/mm^2 (ranging between 0 and 18). When comparing eosinophils in tissues between all LCL and DCL patients, a significant difference was found between the groups, with significantly higher numbers in DCL patients ($p < 0.05$) (Fig. 2). However, when specifically comparing LCL patients with tissue eosinophils to all DCL patients, no significant differences were found between the two groups of patients. LCL patients with tissue eosinophils showed an average of 13 ± 1.6 eosinophils/mm^2 (ranging between 5 and 18), while DCL patients showed an average of 14 ± 1.1 eosinophils/mm^2 (ranging between 11 and 17). Histopathological analysis of infected tissues revealed that DCL patients had loss of tissue integrity and a higher parasite load, compared to those of LCL patients (Fig. 2).

Anti-Leishmania IgE and IgG in Sera from LCL and DCL Patients

Serum levels of IgE and IgG were measured in LCL (n=8) and DCL (n=8) patients to determine the serological response to *L. mexicana*. Both antibodies were identified in all patients, with no significant differences in IgE levels between DCL (1.43 ± 0.18) and LCL patients (0.95 ± 0.19). However, both patient groups produced significantly more IgE than controls (0.167 ± 0.068) (Fig. 3A). When

comparing IgG production, DCL patients (1.73 ± 0.05) produced significantly more than LCL patients (1.31 ± 0.11), and both patient groups produced more IgG than controls (0.293 ± 0.074) (Fig. 3B).

Cytokine (IL-6, IL-13, and IL-8) Production and Oxidative Burst by Eosinophils of LCL and DCL Patients Incubated with L. mexicana Promastigotes

Our data indicate that IL-6 and IL-13 production by eosinophils from patients with DCL (n=7) was significantly higher, compared to those from LCL patients (n=7) or healthy control subjects (n=7), when incubated with *L. mexicana* promastigotes. IL-6 production by DCL eosinophils (45 ± 13.2 pg/mL) was significantly higher compared to LCL patients (7.4 ± 1 pg/mL). Control subjects did not exhibit IL-6 production (Fig. 4A).

IL-8 production by eosinophils of DCL patients (45 ± 2.6 pg/mL) was also significantly higher compared to LCL patients (29 ± 2 pg/mL) and control subjects (40 ± 1.7 pg/mL). Interestingly, eosinophils of control subjects produced nearly the same amount of IL-8 as those of DCL patients) (Fig. 4B).

In the case of IL-13 production, eosinophils of DCL patients showed a significantly higher production (1000 ± 11.5 pg/mL) compared to LCL patients and controls (50 ± 2.8 pg/mL) (Fig. 4C). These findings demonstrate that eosinophils from DCL patients produce significantly more IL-6, IL-8, and IL-13 when exposed to *L. mexicana* promastigotes compared to those of LCL patients. Unstimulated

eosinophils did not exhibit IL-6, IL-8, and I-13 cytokine production and were below the ELISA cut-off point.

The analysis of the oxidative burst in eosinophils of LCL (n=7), DCL (n=7), and healthy subjects (n=7) co-incubated with *L. mexicana* promastigotes showed that the parasite enhances the magnitude of the oxidative burst in eosinophils of both groups of patients to an equal extent, which was significantly higher compared to eosinophils form healthy controls (Fig. 4D). However, no significant differences between both clinical forms.

Phagocytosis and Lysis of Leishmania mexicana Promastigotes by Eosinophils

Microscopic analysis revealed that purified eosinophils do not release granules in the absence of parasites (Fig. 5A). When co-incubated with *L. mexicana,* eosinophils exhibited the ability to damage extracellular promastigotes by releasing cytoplasmic granules. This damaged resulted in the alteration of parasite morphology and the acquisition of a deformed phenotype (Fig. 5B). Giemsa staining further demonstrated that damage occurred when *Leishmania* was in close contact with the eosinophils, as well as when parasites were surrounded by clouds of massively released granules (Fig. 5B). Staining with CFDA revealed that *L. mexicana* promastigotes began to form clusters in the culture medium (Fig. 5C). After incubation with eosinophils, the promastigotes are phagocytosed and transformed into intracellular amastigotes, ultimately leading to their degradation (Fig. 5D). The percentage of eosinophil degranulation was significantly higher in eosinophils co-incubated with *Leishmania* (Fig.5e).

Eosinophils Release Cytoplasmic "Degranulation Sacs" with Granules in Response to Leishmania

Co-incubation of eosinophils with *L. mexicana* revealed that, in addition to diffusely releasing cytoplasmic granules near parasites, eosinophils also release cytoplasmic "degranulation sacs" containing granules with electron-dense characteristics, surrounded by a membrane (Fig. 6). Subsequently, as the cell membrane was lost, individual granules came into close contact with the parasites, leading to damage (Figs. 6A and 6B). Electron microscopy analysis further illustrated that eosinophils could shed substantial portions of their cytoplasm, including granules, in the form of "degranulation sacs" onto the parasite. This phenomenon was evidenced by the presence of submembrane microtubules and caused significant damage to the parasites. Signs of parasite damage included diffuse osmophilic areas, indicating damage to intracellular membranes (Figs. 6C and 6D).

Extracellular Traps are Induced by L. mexicana

Another leishmanicidal mechanism observed *in vitro* was the release of extracellular traps (EETs) by eosinophils, which entrapped the parasites and brought them into contact with released granules such as MPB (7A). Under resting conditions, only eosinophil nuclear DNA was stained blue (7B). However, after coincubation with *Leishmania,* eosinophils released nets containing DNA, as indicated by blue DAPI staining, which binds DNA (Fig. 7C). These DNA nets effectively trapped the parasites (Fig. 7D).

Discussion

Eosinophils, constituting 2-5% of human blood leukocytes, play a multifunctional role in defending against various infections, including parasites, viruses, bacteria, and fungi [5, 6, 16]. However, Yet, the understanding of eosinophils' role in patients infected with *L. Mexicana,* presenting with varying disease severity, such as localized (LCL) or diffuse (DCL) cutaneous leishmaniasis, remains limited.

In our study, we observed that all DCL patients, and a subset of LCL patients with prolonged disease evolution exhibited both blood and tissue eosinophilia. Blood eosinophilia has previously been reported in *L. mexicana*-infected DCL patients and recently cured LCL patients [17], as well as in patients infected with high numbers of *L. donovani* parasites [18]. Mouse models of leishmaniasis have also demonstrated that the eosinophil responses depend on mouse strains, *Leishmania* species, and the phase of infection [11, 19, 20]. Elevated eosinophil numbers, persisting throughout the infection, have been described in susceptible mouse strains infected with *L. mexicana* and *L. amazonensis*, which correlated with enhanced tissue damage and high numbers of intracellular amastigotes [7,11].

Eosinophils employ various mechanisms to control pathogens, including oxidative bursts, phagocytosis, and the release of eosinophil extracellular traps (EETs). EETs contain histones, eosinophil cationic proteins, and cytotoxic granules such as MBP [21-22] within "degranulation sacs", which are released through a single fusion pore [23]. Furthermore, the shedding of clusters of free extracellular eosinophil granules, as well as of plasma membrane-enveloped structures containing cytoplasmic granules, has been reported. After membrane lysis, the granules are released into the extracellular space, where they function as free

granules [24].

Our findings demonstrate that eosinophils phagocytize *L. mexicana* promastigotes *in vitro* (Supplementary 1), which is accompanied by various forms of granule exocytosis that inflict damage to the parasite, as confirmed by electron microscopy and Giemsa staining. The contact between the parasites and eosinophils induced the release of free granules and the extrusion of membrane bound “degranulation sacs" containing numerous granules, which are released near the parasites, leading to damage (Supplementary 2). This phenomenon resembles a piecemeal degranulation process [23, 24]. Furthermore, eosinophils released EETs containing MBP in response to *L. mexicana* parasites. Our data align with previous reports of eosinophils attaching to and phagocytizing *Leishmania mexicana amazonensis* parasites, resulting in oxidative bursts and the release of extracellular traps containing membrane-bound clusters of granules, as well es free granules, both of which potentially contribute to parasite elimination [25-27]. Although our observations were conducted *in vitro,* this phenomenon may be expected to occur in infected tissues, thus contributing to tissue damage [28, 29].

Eosinophil cytokines modulate the immune response, but their involvement in defining the virulence of cutaneous leishmaniasis is not well understood. Leishmaniasis, a disease with divergent outcomes based on the balance of pro- and anti-inflammatory cytokines, is regulated by cytokine-producing cells. Th1 cytokines support parasite control, while a Th2 response is associated with susceptibility [6, 16]. Given that the immune response in DCL patients cannot effectively control the extensive spread of parasites, we were interested in examining whether eosinophil responses to *L. mexicana* differ between DCL and

LCL patients, potentially favoring disease progression.

Our data now show that eosinophils from DCL patients secrete significantly higher levels of IL-6, IL-8 and IL-13 in response to *L. mexicana* promastigotes, compared to eosinophils from LCL patients. This differential response between DCL and LCL eosinophils, may be related to the genetic background of patients, as previous studies have reported differential expression of inflammatory genes in NK cells from DCL and LCL patients following TLR stimulation with LPG, with LCL NK cells expressing more genes associated to inflammatory responses compared to DCL NK cells [30].

Previous studies have reported the presence of IL-6 during the active phases of infection by several *Leishmania* species [31- 33]. IL-6 along with IL-1 and IL-23 regulates Th17 cell differentiation [33]. The Th17 response promotes the chemotaxis of neutrophils through the induction of IL-8 on fibroblasts, epithelial, and endothelial cells [34]. In experimental cutaneous leishmaniasis, the Th17 response has been associated with an increase in pathological conditions, primarily due to the neutrophil-mediated promotion of Th17 response [35].

Early recruitment of neutrophils to the infection site mediated by IL-8 also leads to their activation and enhanced phagocytosis of the parasite. Neutrophils not only harbor the parasite, shielding it from the deleterious effects of complement, but they have also been described to aid in parasite spread through “silent” phagocytosis that occurs after apoptotic cells containing the parasites are phagocytosed by nonactivated macrophages (“Trojan horse” strategy) [36-38]. Our results of enhanced production of this chemokine are in line with the literature, where infections by *L. infantum,* and *L. major* have all been shown shown to

enhance the production of IL-8 [39-41]. Additionally, aside from disease modulation resulting from enhanced IL-8 production, polymorphisms in IL-8 (−251A/T) or IL-6 (174G/C) genes should also be considered in future studies, as they have been shown to influence the outcome of mucosal leishmaniasis [42-44]. This aspect remains to be analyzed in cutaneous leishmaniasis caused by *L. mexicana.*

Our finding of elevated production of IL-13 by eosinophils of DCL patients is another factor that might contribute to enhanced disease severity. Il-13 activates eosinophils and promotes the development of alternatively activated macrophages, which have been shown to promote disease progression in cutaneous Leishmaniasis [16, 33, 36]. This cytokine is functionally redundant with IL-4 and inhibits the production of inflammatory cytokines, thereby interfering with resistance to *Leishmania* [45]. Furthermore, IL-13 can promote the isotype switching of B cells to produce IgE and IgG4 in humans [46]. Our observations reveal that both DCL and LCL patients have elevated anti-*Leishmania* IgE and IgG antibodies. Although no differences were observed in IgE production between both clinical forms, it is noteworthy that both patient groups tested positive for IgE, contrasting with other reports of IgE in *L. amazonensis* infections, where only 40% of patients were positive for this antibody isotype [47]. However, we found that DCL patients had significantly higher levels of IgG, compared to LCL patients. This is noteworthy, as higher amounts of IgG in leishmaniasis have been associated with susceptibility, as IgG induces the production of regulatory IL-10 in monocytes, contributing to the inhibition of cellular responses in chronic DCL patients [48].

In summary, these three cytokines have been associated with disease susceptibility, and our data align with the literature, showing their enhanced

production by eosinophils from DCL patients under *in vitro* conditions. It is possible the higher eosinophil numbers in the infiltrated inflammatory areas of DCL patients contribute to the production of these cytokines, further exacerbating disease severity. However, this hypothesis remains to be investigated in the context of cutaneous leishmaniasis caused by *L. mexicana.*

Our results now shed new light on the apparent dual role of eosinophils in leishmaniasis. They can inflict crucial damage to the parasite, and produce cytokines such as IL-6, IL-8 and IL-13 upon contact with the parasite, which are associated with disease severity and pathological conditions.

Conclusions

Eosinophils employ diverse effector mechanisms when co-incubated with *Leishmania mexicana,* including the exocytosis of granules onto the parasite, the release of membrane bound "degranulation sacs", and the formation of EETs, all of which contribute to parasite damage. However, despite presenting eosinophilia, DCL patients exhibit a high parasite load and uncontrolled disease progression. Given that eosinophils from DCL patients also release significantly higher amounts of cytokines IL-6, IL-13, and the chemokine IL-8, compared to LCL patients, these mediators may contribute to the disease progression observed in DCL patients. Our data provide additional insights into the innate defense mechanisms exerted by eosinophils against *Leishmania mexicana*. To further clarify the biological significance of eosinophils in leishmaniasis, it would be interesting to study other effector mechanisms, including signaling pathways, signal transductions, and polymorphisms in the eosinophils of these patients, in order to enhance our understanding of eosinophils in this disease.

Conflict of Interest

We declare that this work has not been influenced by any financial, personal, or professional interest.

Author Contributions

Conceptualization: NSS, RPA, JDD, APT and IB

Formal Analysis: ICCG, JZC, JDD, HARM

Funding Acquisition: IB

Investigation GCF, NSS, ICCG, APT, JZC, VJLA, BSB, ARR, JDD, RPA and RBCS.

Methodology: NSS, APT, ARR, JDD and RPA

Project Administration: IB

Resources**:** BSB, VJLA, GCF

Supervision: IB

Visualization: GCF, NSS, ICCG, APT, JZC, VJLA, BSB and RBCS.

Writing – Original Draft Preparation: RPA and NSS

Writing – Review & Editing: ICCG, APT, JZC and IB

Funding Statement

The funders had no role in study design, data collection and analysis, decision to publish or preparation of the manuscript.

References

1. Scorza BM, Carvalho EM, Wilson ME. Cutaneous Manifestations of Human and Murine Leishmaniasis. Int J Mol Sci. 2017 Jun 18;18(6):1296.

2. Gabriel Á, Valério-Bolas A, Palma-Marques J, Mourata-Gonçalves P, Ruas P, Dias-Guerreiro T, et al. Cutaneous Leishmaniasis: The Complexity of Host's Effective Immune Response against a Polymorphic Parasitic Disease. J Immunol Res. 2019 Dec 1;2019:2603730.

3. Leishmaniases. Epidemiological report on the region of the Americas. Pan American Health Organization. No. 11 Dec 2022. Consulted January 2023. Available from: https://iris.paho.org/bitstream/handle/10665.2/56831/PAHOCDEVT220021_eng.pdf?sequence=1&isAllowed=y

4. Conceição-Silva F, Reis CSM, De Luca PM, Leite-Silva J, Santiago MA, Morrot A, et al. The Immune System Throws Its Traps: Cells and Their Extracellular Traps in Disease and Protection. Cells. 2021 Jul 26;10(8):1891.

5. Klion AD, Ackerman SJ, Bochner BS. Contributions of Eosinophils to Human Health and Disease. Annu Rev Pathol. 2020 Jan 24;15:179-209.

6. Ramirez GA, Yacoub MR, Ripa M, Mannina D, Cariddi A, Saporiti N, et al. Eosinophils from Physiology to Disease: A Comprehensive Review. Biomed Res Int. 2018 Jan 28;2018:9095275.

7. Grimaldi G Jr, Soares MJ, Moriearty PL. Tissue eosinophilia and *Leishmania mexicana mexicana* eosinophil interactions in murine cutaneous leishmaniasis. Parasite Immunol. 1984 Sep;6(5):397-408.

8. Bittencourt AL, Barral A, Costa JM, Saldanha AC, Badaró F, Barral-Netto M, et al. Diffuse cutaneous leishmaniasis with atypical aspects. Int J Dermatol. 1992 Aug;31(8):568-70.

9. Cáceres-Dittmar G, Tapia FJ, Sánchez MA, Yamamura M, Uyemura K, Modlin RL, et al. Determination of the cytokine profile in American cutaneous leishmaniasis using the polymerase chain reaction. Clin Exp Immunol. 1993 Mar;91(3):500-5.

10. Saito S, Hamada A, Watanabe N, Obata T, Katakura K, Ohtomo H. Eosinophil chemotactic activity in *Leishmania amazonensis* promastigotes. Parasitol Res. 1996;82(6):485-9.

11. de Oliveira Cardoso F, de Souza Cda S, Mendes VG, Abreu-Silva AL, Gonçalves da Costa SC, Calabrese KS. Immunopathological studies of *Leishmania amazonensis* infection in resistant and in susceptible mice. J Infect Dis. 2010 Jun 15;201(12):1933-40.

12. Watanabe Y, Hamaguchi-Tsuru E, Morimoto N, Nishio Y, Yagyu K, Konishi Y, et al. IL-5-Induced Eosinophils Suppress the Growth of *Leishmania amazonensis* In Vivo and Kill Promastigotes In Vitro in Response to Either IL-4 or IFN-gamma. DNA Cell Biol. 2004 Jul;23(7):412-8.

13. Salaiza-Suazo N, Volkow P, Tamayo R, Moll H, Gillitzer R, Pérez-Torres A, et al. Treatment of two patients with diffuse cutaneous leishmaniasis caused by *Leishmania mexicana* modifies the immunohistological profile but not the disease outcome. Trop Med Int Health. 1999 Dec;4(12):801-11.

14. Kovács I, Horváth M, Kovács T, Somogyi K, Tretter L, Geiszt M, et al. Comparison of proton channel, phagocyte oxidase, and respiratory burst levels between human eosinophil and neutrophil granulocytes. Free Radic Res. 2014 Oct;48(10):1190-9.

15. Real F, Pouchelet M, Rabinovitch M. *Leishmania* (*L.*) *amazonensis*: fusion between parasitophorous vacuoles in infected bone-marrow derived mouse macrophages. Exp Parasitol. 2008 May;119(1):15-23.

16. Ariyaratne A, Finney CAM. Eosinophils and Macrophages within the Th2-Induced Granuloma: Balancing Killing and Healing in a Tight Space. Infect Immun. 2019 Sep 19;87(10):e00127-19.

17. Lezama-Dávila CM, Isaac-Márquez AP, Padierna-Olivos J, Aguilar-Torrentera F, Chapa-Ruiz R. Immunomodulation of Chiclero's ulcer. Role of eosinophils, T cells, tumour necrosis factor and interleukin-2. Scand J Immunol. 1998 May;47(5):502-8.

18. Wijesinghe H, Gunathilaka N, Semege S, Pathirana N, Manamperi N, de Silva C, et al. Histopathology of Cutaneous Leishmaniasis Caused by *Leishmania donovani* in Sri Lanka. Biomed Res Int. 2020 May 2;2020:4926819.

19. Monteiro MC, Lima HC, Souza AA, Titus RG, Romão PR, Cunha FQ. Effect of *Lutzomyia longipalpis* salivary gland extracts on leukocyte migration induced by *Leishmania major*. Am J Trop Med Hyg. 2007 Jan;76(1):88-94.

20. Figueredo LA, de Paiva-Cavalcanti M, Almeida EL, Brandão-Filho SP, Dantas-Torres F. Clinical and hematological findings in *Leishmania braziliensis*-infected dogs from Pernambuco, Brazil. Rev Bras Parasitol Vet. 2012 Oct-Dec;21(4):418-20.

21. Williams TL, Rada B, Tandon E, Gestal MC. "NETs and EETs, a Whole Web of Mess". Microorganisms. 2020 Dec 4;8(12):1925.

22. Soragni A, Yousefi S, Stoeckle C, Soriaga AB, Sawaya MR, Kozlowski E, et al. Toxicity of eosinophil MBP is repressed by intracellular crystallization and promoted by extracellular aggregation. Mol Cell. 2015 Mar 19;57(6):1011-1021.

23. Crivellato E, Nico B, Mallardi F, Beltrami CA, Ribatti D. Piecemeal degranulation as a general secretory mechanism? Anat Rec A Discov Mol Cell Evol Biol. 2003 Sep;274(1):778-84.

24. Fettrelet T, Gigon L, Karaulov A, Yousefi S, Simon HU. The Enigma of Eosinophil Degranulation. Int J Mol Sci. 2021 Jun 30;22(13):7091. doi: 10.3390/ijms22137091.

25. Pimenta PF, Dos Santos MA, De Souza W. Fine structure and cytochemistry of the interaction between *Leishmania mexicana amazonensis* and rat neutrophils and eosinophils. J Submicrosc Cytol. 1987 Jul;19(3):387-95.

26. Ueki S, Melo RC, Ghiran I, Spencer LA, Dvorak AM, Weller PF. Eosinophil extracellular DNA trap cell death mediates lytic release of free secretion-competent eosinophil granules in humans. Blood. 2013 Mar 14;121(11):2074-83.

27. Guimarães-Costa AB, Nascimento MT, Froment GS, Soares RP, Morgado FN, Conceição-Silva F, et al. *Leishmania amazonensis* promastigotes induce and

are killed by neutrophil extracellular traps. Proc Natl Acad Sci U S A. 2009 Apr 21;106(16):6748-53.

28. Rodríguez NE, Wilson ME. Eosinophils and mast cells in leishmaniasis. Immunol Res. 2014 Aug;59(1-3):129-41.

29. Nija RJ, Sanju S, Sidharthan N, Mony U. Extracellular Trap by Blood Cells: Clinical Implications. Tissue Eng Regen Med. 2020 Apr;17(2):141-153.

30. Fernández-Figueroa EA, Imaz-Rosshandler I, Castillo-Fernández JE, Miranda-Ortíz H, Fernández-López JC, Becker I et al. (2016). Down-Regulation of TLR and JAK/STAT Pathway Genes Is Associated with Diffuse Cutaneous Leishmaniasis: A Gene Expression Analysis in NK Cells from Patients Infected with *Leishmania mexicana*. PLoS Negl Trop Dis. 2016 Mar 31; 10(3): e0004570.

31. de Lima VM, Peiro JR, de Oliveira Vasconcelos R. IL-6 and TNF-alpha production during active canine visceral leishmaniasis. Vet Immunol Immunopathol. 2007 Jan 15;115(1-2):189-93.

32. Dos Santos PL, de Oliveira FA, Santos ML, Cunha LC, Lino MT, de Oliveira MF, et al. The Severity of Visceral Leishmaniasis Correlates with Elevated Levels of Serum IL-6, IL-27 and sCD14. PLoS Negl Trop Dis. 2016 Jan 27;10(1):e0004375.

33. Maspi N, Abdoli A, Ghaffarifar F. Pro- and anti-inflammatory cytokines in cutaneous leishmaniasis: a review. Pathog Glob Health. 2016 Sep;110(6):247-260.

34. Huang N, Dong H, Luo Y, Shao B. Th17 Cells in Periodontitis and Its Regulation by A20. Front Immunol. 2021 Sep 7; 12:742925.

35. Pedraza-Zamora CP, Delgado-Domínguez J, Zamora-Chimal J, Becker I. Th17 cells and neutrophils: Close collaborators in chronic *Leishmania mexicana* infections leading to disease severity. Parasite Immunol. 2017 Apr;39(4).

36. Dayakar A, Chandrasekaran S, Kuchipudi SV, Kalangi SK. Cytokines: Key Determinants of Resistance or Disease Progression in Visceral Leishmaniasis: Opportunities for Novel Diagnostics and Immunotherapy. Front Immunol. 2019 Apr 5;10:670.

37. Beste MT, Lomakina EB, Hammer DA, Waugh RE. Immobilized IL-8 Triggers Phagocytosis and Dynamic Changes in Membrane Microtopology in Human Neutrophils. Ann Biomed Eng. 2015 Sep;43(9):2207-19.

38. Carreira, JC, da Silva AV. The Role of Neutrophils in the Interaction with *Leishmania*: Far beyond a Simple Trojan Horse?. Open J. Anim. Sci. 2021 Jun 25; 11, 399-421.

39. Badolato R, Sacks DL, Savoia D, Musso T. *Leishmania major*: infection of human monocytes induces expression of IL-8 and MCAF. Exp Parasitol. 1996 Jan;82(1):21-6.

40. Boussoffara T, Boubaker MS, Ben Ahmed M, Mokni M, Guizani I, Ben Salah A, et al. Histological and immunological differences between zoonotic cutaneous leishmaniasis due to *Leishmania major* and sporadic cutaneous leishmaniasis due to *Leishmania infantum*. Parasite. 2019;26:9.

41. Oualha R, Barhoumi M, Marzouki S, Harigua-Souiai E, Ben Ahmed M, Guizani I. Infection of Human Neutrophils With *Leishmania infantum* or *Leishmania major* Strains Triggers Activation and Differential Cytokines Release. Front Cell Infect Microbiol. 2019 May 10;9:153.

42. Hajilooi M, Abasi M, Bazmani A, Ahmadi A, Matini M, Solgi G, et al. Evaluation of interleukin-8 -251 t/a polymorphisms in visceral leishmaniasis. J Res Health Sci. 2015 Winter;15(1):59-61.

43. Castellucci L, Menezes E, Oliveira J, Magalhaes A, Guimaraes LH, Lessa M, et al. IL6 -174 G/C promoter polymorphism influences susceptibility to mucosal but not localized cutaneous leishmaniasis in Brazil. J Infect Dis. 2006 Aug 15;194(4):519-27.

44. Cardoso T, Bezerra C, Medina LS, Ramasawmy R, Scheriefer A, Bacellar O, et al. *Leishmania braziliensis* isolated from disseminated leishmaniasis patients downmodulate neutrophil function. Parasite Immunol. 2019 May;41(5):e12620.

45. Zaatar MT, Simaan Y, Karam MC. Exogenous IL-13 exacerbates *Leishmania major* infection and abrogates acquired immunity to re-infection. Parasitol Res 2022 Jul;121(7):2009-2017.

46. Le Floc'h A, Allinne J, Nagashima K, Scott G, Birchard D, Asrat S, et al. Dual blockade of IL-4 and IL-13 with dupilumab, an IL-4Rα antibody, is required to broadly inhibit type 2 inflammation. Allergy. 2020 May;75(5):1188-1204.

47. Sousa-Atta ML, Salamé GS, D'Oliveira A Jr, Almeida RP, Atta AM, Carvalho EM. Immunoglobulin E antileishmanial antibody response in cutaneous leishmaniasis. Clin Diagn Lab Immunol. 2002 Jan;9(1):101-4.

48. Buxbaum LU. *Leishmania mexicana* infection induces IgG to parasite surface glycoinositol phospholipids that can induce IL-10 in mice and humans. PLoS Negl Trop Dis. 2013 May 9;7(5):e2224.

Figure legends

Figure 1. Eosinophils in Peripheral Blood of Patients with Cutaneous Leishmaniasis.

A. Percentage of blood eosinophils from patients with LCL (green circles, n = 30) and DCL (red squares, n = 4). B. Blood eosinophils and disease duration. The horizontal line represents the mean, and each dot represents a patient (female or male). Bars represent the mean ± SEM. ns: non-significant.

Figure 2. Eosinophils in Lesions of Patients with Cutaneous Leishmaniasis.

Eosinophils in the tissue were quantitated and expressed as eosinophils/mm^2. The bar graph shows the mean, and each dot represents a patient. Representative H&E staining of biopsies taken from LCL patients (green circles, n=35) and DCL patients (red squares, n=5) are shown. Black arrows indicate *Leishmania* amastigotes and blue arrows indicate eosinophils. Scale bar = 20 µm. Bars represent the mean ± SEM, and asterisks represent statistically significant differences ($P < 0.05$).

Figure 3. Anti-*Leishmania* IgE and IgG in Sera from Patients with Cutaneous Leishmaniasis.

A. IgE levels. B. IgG levels. Each symbol represents a patient, and the horizontal line represents the mean of each group. LCL (n=8) and DCL (n=8) patients, healthy controls (n=8). *All symbols represent statistically significant differences (P < 0.05).

Figure 4. Cytokine Production and Oxidative Burst were Analyzed in Eosinophils from Patients with LCL and DCL.

Eosinophils from LCL and DCL patients and from healthy controls were incubated with *L. mexicana* promastigotes. A. IL-6 production. B. IL-8 production. C. IL-13 production. D. ROS production measured by luminol reaction (in mV). Bars represent the mean ± SEM (n= 7 for each group of patients and healthy control subjects). All symbols represent statistically significant differences (P < 0.05).

Figure 5. Phagocytosis and Lysis of *Leishmania mexicana* by Eosinophils.

A. Purified eosinophils in the absence of parasites. B. Eosinophils incubated with *L. mexicana* promastigotes (Giemsa stained). Close contact between parasites and cells leads to abundant degranulation (green arrow) and damaged parasites (black arrows). C. *Leishmania* promastigotes stained with CFDA. D. Eosinophils co-incubated with *Leishmania* promastigotes show phagocytosed parasite (black arrow). Scale bar = 20 µm. E. Percentage of eosinophil degranulation with or without *L. mexicana* (n=3). Bars represent the mean ± SD, and the asterisk represents statistically significant differences (P <0.05).

Figure 6. Release of Cytoplasmic “Degranulation Sacs” by Eosinophils Co-incubated with Parasites.

A. Eosinophil (green arrow) and release of cytoplasmic “degranulation sacs". B. Degranulation sacs (black arrows) and parasites (pink arrow). C and D: Ultramicroscopic images showing released specific or secondary granules (g) or within cytoplasmic pockets limited by an intact membrane (black arrows). These “degranulation sacs” were in close apposition to distorted *L. mexicana* (Lm). Parasites, identified by their submembranous microtubule cytoskeleton (arrowheads) show signs of damage as evidenced by osmiophilic areas (white asterisk), nuclear fragmentation (n), and a clear or aqueous cytoplasm denoting an extensive cytoplasmic vacuolization. (D) Some eosinophil specific granules still retain the characteristic electron-dense central crystalline core, surrounded by a clearer peripheral matrix (red arrows). Light microscopy scale bar = 20 µm.

Figure 7. Extracellular Eosinophil Traps Induced by Co-incubation with *L. mexicana*.

A) Extracellular traps (black asterisk) containing MBP-positive granules (black arrow) and parasites within the nets (red arrow). B) Purified eosinophils show their nuclei stained blue with DAPI (green arrow). No nets are formed in the absence of *Leishmania.* C) Eosinophils co-incubated with *L. mexicana* promastigotes show the formation of extracellular DNA traps (white arrow). D) The same image (C) was analyzed with light and fluorescence microscopy, showing the parasites (red arrow) in contact with nets (white arrow). Scale bar = 20 µm.

Supplementary 1. Phagocytosed Parasites by Eosinophils.

A) Parasites stained with CFDA (green color) phagocytosed by eosinophils are observed inside these cells. Black arrows show phagocytized parasites. B) Remains of degraded parasites (black arrows) are observed already inside the eosinophils (green color granules).

Supplementary 2. *Leismania mexinana* Damaged by Eosinophil Contact.

A) Normal shape and size of a viable *Leishmania* promastigote. B) Morphological changes in parasite size, damage to membranes, formation of small vacuoles within the parasite, and loss of flagellum were observed in parasites co-incubated with eosinophils in a 1:10 ratio for 1hour. Black arrows show damaged parasites.

Reviewer #1: The article “Eosinophils of patients with localized and diffuse cutaneous leishmaniasis: differential response to Leishmania mexicana, with insights into mechanisms of damage inflicted upon the parasites by eosinophils “ by Norma Salaiza-Suazo et al., describe in several experiments the presence and response of eosinophils in LCL and DCL. Eosinophils have been neglected in the leishmaniasis contexts, probably because their significance is overshadowed by the importance of macrophages and T cells. This study address a relevant gap in the knowledge. However results here shown are rather descriptive than conclusive.

Major comments:

1) Methods section is in many points poorly described:
a) Need more specification on the eosinophil isolation: How long was centrifuged? How was done the RBC lysis? How many washes/time/g-force?

R= New information was added on lines 168-190

b) -Leishmania was cultured in the absence of antibiotics?
R=The information was added on lines 196-197.

c) -Also, in-house method for IL-8 measurement lacks important information: which capture/detection antibody was used? Please describe the protocol.

R=The information was added on lines 207-226.

d) -How was the cut-off in ELISA calculated?

R= The cut-off point information was added on lines 223-224.

e) - How was the cytometry done? What was the antibodies used and gating strategy applied?

R= Eosinophils in peripheral blood were determined by FCS and SSC by flow cytometry, this information was added on lines 133-134. The figure below represents the gating strategy.

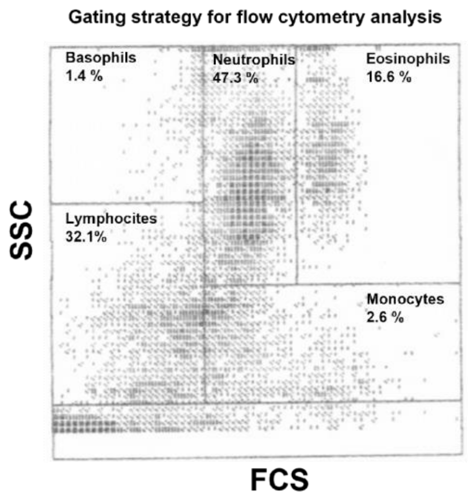

f) -Why is the luminescence in oxidative bursts measured as Mmvolt?

R= The Luminoskan's light detector has a photomultiplier tube (photomultiplier tube), a signal acquisition and processor system, which are in a closed compartment, where all the chemiluminescence is captured by the photomultiplier tube which sends the light signal and according to the configuration of our device (Luminoskan) the maximum light signal generated in the chemiluminescence reaction is reported in mVolts.

Line 309 – 310 "These findings demonstrate that eosinophils from patients with DCL produce significantly more IL-6, IL-8, and IL-13 when exposed to L. mexicana promastigotes, compared to those of LCL patients." Cytokine measurement should have been done with unstimulated eosinophils. This is important to be sure that the cytokine production is not due to a pre-activated status. How could the authors be sure the cytokines produced were induced by parasites rather than being already activated?

R= Unstimulated eosinophils were included in all cytokine production determinations. However, they were not included in the plot because they were below the cut-off point. The information was added on lines 352-354.

Line 318-330 – To assume that eosinophils are being harmful against leishmania, the authors should show quantitatively increased death of promastigotes while in contact with Leishmania. They could use cellular death markers (Propidium iodide for example) to quantify the number of dead promastigotes in contact with eosinophil.

R= No staining with supravital dyes was performed, however, to demonstrate cell damage we are now including a new supplementary figure 2 showing Giemsa-stained viable intact parasites compared to damaged parasites incubated with eosinophils, where morphological changes and cell damage of the parasites are visible. This observation

was also evidenced in the electron microscopy photographs of Figure 6, showing damage of the external membrane of the parasite.

Lines 407-409 – It is not correct to associate IL-6 with TH2 responses. IL-6 is mainly produced by myeloid cells (mostly macrophages) and this cytokine is increased during VL together with a TH2 response, independently. IL-6 is required to a TH17 response and whether their production by eosinophils is important/significant during cutaneous leishmaniasis is yet to be shown.

R= We agree and have removed the statement regarding the association of IL-6 with a Th2 response. Instead, we added the relevance of IL-6 in the Th17 response in cutaneous leishmaniasis on lines 459-464.

Line 435: The authors says that “it is noteworthy that 70% LCL and 90% DCL were positive for IgE”, although figure 3 clearly shows (as well as is described in the text) that all patients were IgG and IgE positive. Please revise this excerpt and figure.

R= We thank the reviewer and made the correction on line 488.

Lines 442 – 444 – authors must be careful to assume “an enhanced production of cytokines” in DCL patients, since they only measured eosinophil derived cytokines and this does not mean that these cytokines are in fact increased in this patients or even related to susceptibility (for example, IFN-g is associated with resistance and it is also increased during VL). The production of these cytokines by eosinophils compared to other cells (macrophages and monocytes) might be insignificant in natural conditions.

R= We thank the reviewer and have changed the text on lines 496-501.

Figure 5 - Authors claim to demonstrate phagocitozed leishmania by eosinophil but the image has bad quality. Do authors have clear evidence of phagocytosis by eosinophils (stained slides would be enough).

R= Figure 5 was modified with a better photograph in panel 5D that shows parasites phagocyted by eosinophils. Additionally, we added a new supplementary figure 1 with more photographs showing the phagocytosis and possible intracellular degradation of the parasite.

Authors should discuss why an innate immune cell would respond differently in DCL and LCL. Which possible mechanisms would allow eos from DCL to be more prone to respond to promastigotes than LCL?

R= We now added a possible explanation for the differential response between LCL and DCL patients on lines 452-457, including a new reference (30) where differences in genetic background between both patients have been reported.

Line 449: “their release of the cytokines IL-6, IL-8 and IL-13 promotes disease severity”: the data here shown do not support this statement.

R= We agree with the reviewer and changed the statement on lines 503-505.

Reviewer #2: The authors report on an important and up-to-now largely neglected aspect of a cellular repsonse to Leishmania infection. This study certainly will be of great interest.

Major concerns:

- The statistical method employed should be detailled in the respective Fig. legends. The authors might want to use multivariate testing.

R= The objective of the work was to compare the response between eosinophils of LCL and DCL patients and for this approach we decided use to the Mann–Whitney U-test. New information was added in Figure legends 1, 2 and 5, detailing the statistical significance of the figures.

- The Fig. 5 - 7 should be analyzed using a readout that allows statistical analysis. Otherwise these findings seem very anectodal.

R= We agree and modified Figure 5, adding the panel 5E, that quantitatively describes the % of degranulation induced by parasite incubation. This result was also included in line 375-376.

- The authors should demonstrate a by FACS that their MACS-based enrichment of eosinophils worked.

R= We don´t have a FACS analysis of the enrichment of eosinophils, but include for the reviewer the figure below that shows a Giemsa stain of peripheral blood cells from patients before (A, B) and after the MACS-based enrichment (C, D). The purity of eosinophils was >99%. The information was described on lines 190-191. This purity was analyzed by a microscopical analysis of the characteristic acidophilic granules and the polymorphic nuclei as markers of eosinophils.

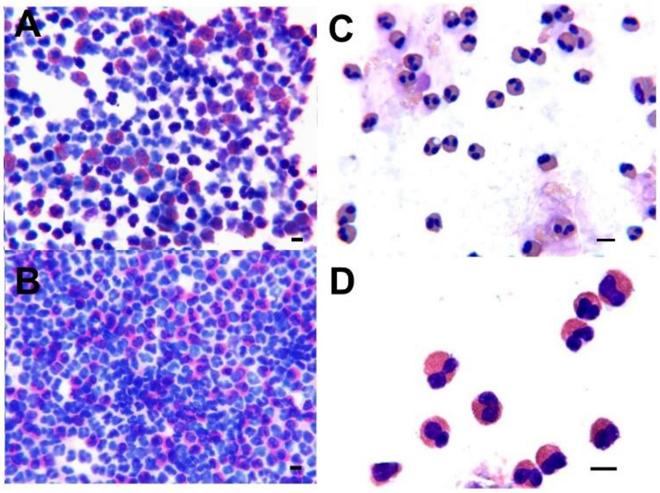

Figure 1. Giemsa stain of eosinophil MACS-based enrichment. A and B show peripherical blood cells from eosinophilic patients. C and D show eosinophil enrichment. Scale bar = 20 µm.
