# Supplementary material for: Eosinophils of patients with localized and diffuse cutaneous leishmaniasis: Differential response to Leishmania mexicana, with insights into mechanisms of damage inflicted upon the parasites by eosinophils
Source: PLoS One. 2024 Feb 15;19(2):e0296887. doi: 10.1371/journal.pone.0296887 (PMC10868813; doi:10.1371/journal.pone.0296887)
Supplement: Attachment — Submitted filename: Response to reviewers.docx [file pone.0296887.s003.docx]

**Reviewer #1:** The article “Eosinophils of patients with localized and diffuse cutaneous leishmaniasis: differential response to Leishmania mexicana, with insights into mechanisms of damage inflicted upon the parasites by eosinophils “ by Norma Salaiza-Suazo et al., describe in several experiments the presence and response of eosinophils in LCL and DCL. Eosinophils have been neglected in the leishmaniasis contexts, probably because their significance is overshadowed by the importance of macrophages and T cells. This study address a relevant gap in the knowledge. However results here shown are rather descriptive than conclusive.

Major comments:

1) Methods section is in many points poorly described:

a) Need more specification on the eosinophil isolation: How long was centrifuged? How was done the RBC lysis? How many washes/time/g-force?

R= New information was added on lines 168-190

b) -Leishmania was cultured in the absence of antibiotics?

R=The information was added on lines 196-197.

c) -Also, in-house method for IL-8 measurement lacks important information: which capture/detection antibody was used? Please describe the protocol.

R=The information was added on lines 207-226.

d) -How was the cut-off in ELISA calculated?

R= The cut-off point information was added on lines 223-224.

e) - How was the cytometry done? What was the antibodies used and gating strategy applied?

R= Eosinophils in peripheral blood were determined by FCS and SSC by flow cytometry, this information was added on lines 133-134. The figure below represents the gating strategy.

f) -Why is the luminescence in oxidative bursts measured as Mmvolt?

R= The Luminoskan's light detector has a photomultiplier tube (photomultiplier tube), a signal acquisition and processor system, which are in a closed compartment, where all the chemiluminescence is captured by the photomultiplier tube which sends the light signal and according to the configuration of our device (Luminoskan) the maximum light signal generated in the chemiluminescence reaction is reported in mVolts.

Line 309 – 310 “These findings demonstrate that eosinophils from patients with
DCL produce significantly more IL-6, IL-8, and IL-13 when exposed to L. mexicana
promastigotes, compared to those of LCL patients.” Cytokine measurement should have been done with unstimulated eosinophils. This is important to be sure that the cytokine production is not due to a pre-activated status. How could the authors be sure the cytokines produced were induced by parasites rather than being already activated?

R= Unstimulated eosinophils were included in all cytokine production determinations. However, they were not included in the plot because they were below the cut-off point. The information was added on lines 352-354.

Line 318-330 – To assume that eosinophils are being harmful against leishmania, the authors should show quantitatively increased death of promastigotes while in contact with Leishmania. They could use cellular death markers (Propidium iodide for example) to quantify the number of dead promastigotes in contact with eosinophil.

R= No staining with supravital dyes was performed, however, to demonstrate cell damage we are now including a new supplementary figure 2 showing Giemsa-stained viable intact parasites compared to damaged parasites incubated with eosinophils, where morphological changes and cell damage of the parasites are visible. This observation was also evidenced in the electron microscopy photographs of Figure 6, showing damage of the external membrane of the parasite.

Lines 407-409 – It is not correct to associate IL-6 with TH2 responses. IL-6 is mainly produced by myeloid cells (mostly macrophages) and this cytokine is increased during VL together with a TH2 response, independently. IL-6 is required to a TH17 response and whether their production by eosinophils is important/significant during cutaneous leishmaniasis is yet to be shown.

R= We agree and have removed the statement regarding the association of IL-6 with a Th2 response. Instead, we added the relevance of IL-6 in the Th17 response in cutaneous leishmaniasis on lines 459-464.

Line 435: The authors says that “it is noteworthy that 70% LCL and 90% DCL were positive for IgE”, although figure 3 clearly shows (as well as is described in the text) that all patients were IgG and IgE positive. Please revise this excerpt and figure.

R= We thank the reviewer and made the correction on line 488.

Lines 442 – 444 – authors must be careful to assume “an enhanced production of cytokines” in DCL patients, since they only measured eosinophil derived cytokines and this does not mean that these cytokines are in fact increased in this patients or even related to susceptibility (for example, IFN-g is associated with resistance and it is also increased during VL). The production of these cytokines by eosinophils compared to other cells (macrophages and monocytes) might be insignificant in natural conditions.

R= We thank the reviewer and have changed the text on lines 496-501.

Figure 5 - Authors claim to demonstrate phagocitozed leishmania by eosinophil but the image has bad quality. Do authors have clear evidence of phagocytosis by eosinophils (stained slides would be enough).

R= Figure 5 was modified with a better photograph in panel 5D that shows parasites phagocyted by eosinophils. Additionally, we added a new supplementary figure 1 with more photographs showing the phagocytosis and possible intracellular degradation of the parasite.

Authors should discuss why an innate immune cell would respond differently in DCL and LCL. Which possible mechanisms would allow eos from DCL to be more prone to respond to promastigotes than LCL?

R= We now added a possible explanation for the differential response between LCL and DCL patients on lines 452-457, including a new reference (30) where differences in genetic background between both patients have been reported.

Line 449: “their release of the cytokines IL-6, IL-8 and IL-13 promotes disease severity”: the data here shown do not support this statement.

R= We agree with the reviewer and changed the statement on lines 503-505.

**Reviewer #2:** The authors report on an important and up-to-now largely neglected aspect of a cellular repsonse to Leishmania infection. This study certainly will be of great interest.

Major concerns:

- The statistical method employed should be detailled in the respective Fig. legends. The authors might want to use multivariate testing.

R= The objective of the work was to compare the response between eosinophils of LCL and DCL patients and for this approach we decided use to the Mann–Whitney U-test.

New information was added in Figure legends 1, 2 and 5, detailing the statistical significance of the figures.

- The Fig. 5 - 7 should be analyzed using a readout that allows statistical analysis. Otherwise these findings seem very anectodal.

R= We agree and modified Figure 5, adding the panel 5E, that quantitatively describes the % of degranulation induced by parasite incubation. This result was also included in line 375-376.

- The authors should demonstrate a by FACS that their MACS-based enrichment of eosinophils worked.

R= We don´t have a FACS analysis of the enrichment of eosinophils, but include for the reviewer the figure below that shows a Giemsa stain of peripheral blood cells from patients before (A, B) and after the MACS-based enrichment (C, D). The purity of eosinophils was >99%. The information was described on lines 190-191. This purity was analyzed by a microscopical analysis of the characteristic acidophilic granules and the polymorphic nuclei as markers of eosinophils.

**Figure 1. Giemsa stain of eosinophil MACS-based enrichment.** A and B show peripherical blood cells from eosinophilic patients. C and D show eosinophil enrichment. Scale bar = 20 µm.
