# Supplementary material for: Eosinophils of patients with localized and diffuse cutaneous leishmaniasis: Differential response to Leishmania mexicana, with insights into mechanisms of damage inflicted upon the parasites by eosinophils
Source: PLoS One. 2024 Feb 15;19(2):e0296887. doi: 10.1371/journal.pone.0296887 (PMC10868813; doi:10.1371/journal.pone.0296887)
Supplement: Attachment — Submitted filename: Response to the reviewer.docx [file pone.0296887.s005.docx]

Dear Editor,

we thank the reviewers for the valuable comments that have improved the manuscript. We have made the suggested corrections and addressed the points raised by the reviewer and hope the manuscript is now suitable for publication.

Corrections are listed according to the lines in the original manuscript, which are shown in the parentheses.

1.- (Line 34) extra spaces were removed in line 34.

2.- (Line 98) **“**mexicana” was corrected to lower case in line 97.

3.- (Line 188-190) Font size was corrected lines 189-190.

4.- (Line 244) the text was corrected to “intracellular parasites” in line 375.

5.- (Line 289) The age range of LCL patients was included in line 289.

6.- (Line 296) the correction was made changing 1B to “1A” in line 295.

7.- (Lines 300, 304) New information and 4 new references (49-52) suggested by the reviewer were included. Furthermore, an additional reference (53) on male susceptibility was also included. The new text included in the manuscript is found in lines 508-522.

8.- (Line 306) We have modified figure 2, including a new graph of eosinophil counts in tissues (2B) of males and females with LCL and DCL. Due to the modifications of Figure 2, changes were made in line 308, where the word “Fig. 2A” was inserted and in line 314. Furthermore, the Figure legend for Figure 2 was modified in lines 816-820. Unfortunately, we do not have all the ages of the patients available at the moment.

9.- (Lines 318-320) A text describing results in Figure 2B was included in lines 815-819.

10.- (Line 353) the correction was made in line 354.

11.- (Line 374) The word “amastigote” was substituted for “parasites” in line 375.

12.- (Line 376) The correction was made and now reads Fig 5“E” in line 377.

13.- (Line 402) The correction was made stating “1-5 %” in line 403, ref. #4 is cited.

14.- (Lines 404 and 405) the corrections were made in lines 405 and 406.

15.- (Line 406) The comment was addressed in lines 508-522.

16.- (Line 451)

#1. The observation was addressed in lines 508-522 of the discussion and 4 new references (49-52) suggested by the reviewer were included. Furthermore, an additional reference (53) on male susceptibility was also included. The new text added to the manuscript is found in lines 508-522.

#2. The observation was addressed in the discussion lines 479-483. The corrected Figure 2B now includes information on eosinophilia and male patients with localized or diffuse cutaneous leishmaniasis and 2 new references (43, 44) were added.

17.- (Line 479) The “L” was corrected and now reads IL-13 in line 485

18.- (Line 503) The sentence was corrected in lines 523-527.

19.- (Line 758) Figure Legend 1 was corrected (lines 806-809). With regard to the suggestion of illustrating males vs females in panel 1A, we believe that the information is already presented in panel 1B, and prefer not to overload panel A.

20) (line 760) The word “dot” was substituted by “symbol” in line 810.

21)- (line 769) We have included more information in new Figure 2B.

22) (Line 800) The additional space was eliminated in line 859.

23) (Line 807) The information of the scale bar was included in line 861.

24) (Line 824) The information for supplementary Figure 1 was added in line 878.

25) (Line 830) The information for supplementary Figure 2 was added in line 885-886.

26) The symbols were modified in Fig.1A and the Figure legend was adjusted (lines 806-810).

Thank you very much.
